# High-Level PM2.5/PM10 Exposure Is Associated With Alterations in the Human Pharyngeal Microbiota Composition

**DOI:** 10.3389/fmicb.2019.00054

**Published:** 2019-01-28

**Authors:** Tian Qin, Furong Zhang, Haijian Zhou, Hongyu Ren, Yinju Du, Shengnan Liang, Fei Wang, Lihong Cheng, Xuguang Xie, Aoming Jin, Yangfeng Wu, Jinxing Zhao, Jianguo Xu

**Affiliations:** ^1^State Key Laboratory of Infectious Disease Prevention and Control, National Institute for Communicable Disease Control and Prevention, Collaborative Innovation Center for Diagnosis and Treatment of Infectious Diseases, Chinese Center for Disease Control and Prevention, Beijing, China; ^2^Shanghai Public Health Clinical Center, Shanghai Institute for Emerging and Re-emerging Infectious Diseases, Shanghai, China; ^3^Centre for Disease Control and Prevention of Liaocheng, Liaocheng, China; ^4^Peking University Clinical Research Institute, Beijing, China

**Keywords:** PM2.5/PM10, pharynx, microbiome composition, smoking, respiratory pathogen

## Abstract

Previous studies showed that high concentration of particulate matter (PM) 2.5 and PM10 carried a large number of bacterial and archaeal species, including pathogens and opportunistic pathogens. In this study, pharyngeal swabs from 83 subjects working in an open air farmer’s market were sampled before and after exposure to smog with PM2.5 and PM10 levels up to 200 and 300 μg/m^3^, respectively. Their microbiota were investigated using high-throughput sequencing targeting the V3–V4 regions of the 16S rRNA gene. The genus level phylotypes was increased from 649 to 767 in the post-smog pharyngeal microbiota, of which 142 were new and detected only in the post-smog microbiota. The 142 new genera were traced to sources such as soil, marine, feces, sewage sludge, freshwater, hot springs, and saline lakes. The abundance of the genera *Streptococcus*, *Haemophilus*, *Moraxella*, and *Staphylococcus* increased in the post-smog pharyngeal microbiota. All six alpha diversity indices and principal component analysis showed that the taxonomic composition of the post-smog pharyngeal microbiota was significantly different to that of the pre-smog pharyngeal microbiota. Redundancy analysis showed that the influences of PM2.5/PM10 exposure and smoking on the taxonomic composition of the pharyngeal microbiota were statistically significant (*p* < 0.001). Two days of exposure to high concentrations of PM2.5/PM10 changed the pharyngeal microbiota profiles, which may lead to an increase in respiratory diseases. Wearing masks could reduce the effect of high-level PM2.5/PM10 exposure on the pharyngeal microbiota.

## Introduction

Air pollution has a serious impact on human health, particularly in developing countries undergoing rapid industrialization and urbanization. Exposure to excessive particulate matter (PM) increases the risk of developing various diseases, leading to increased mortality (Schwartz et al., 1996; Correia et al., 2013). Short-term exposure to PM increases the risk of hospital admission for respiratory diseases (Dominici et al., 2006). For a 10 μg/m^3^ increase in the 2-day average PM2.5 concentration, an increase of 2.07% in respiratory admissions was observed (Zanobetti et al., 2009), and a decrease of 10 μg/m^3^ in the concentration of PM2.5 was associated with an increase in mean life expectancy of 0.35 years (Correia et al., 2013). Starting from early January 2013, eastern and northern China have recorded multiple prolonged and severe smog episodes annually, which were characterized by extremely high concentrations of particles smaller than 2.5 μm (PM2.5) and 10 μm (PM10), with daily peaks of concentrations over 200 μg/m^3^. According to the data collected from 2004 to 2008 in 31 provinces in China, the geographical distribution of high PM concentrations correlated with the geographical distribution of respiratory disease mortality (Cao et al., 2017). An epidemiological study conducted from 2011 to 2015 in Jinan city, the capital of Shandong Province in eastern China, showed that severe smog episodes were associated with a 5.87% increase in overall mortality (Zhang et al., 2017).

Fine PM carries unexpectedly high numbers of microorganisms, some of which might be pathogens or opportunistic pathogens that cause respiratory diseases. Using 16S rRNA sequence-based technology, Cao et al. reported that bacteria were the most abundant prokaryotic microorganisms in PM2.5 and PM10 pollutants sampled during severe smog episodes in Beijing China in 2013. Up to 1315 bacterial and archaeal species were identified. Over 85% of the bacteria carried by PM2.5 and PM10 possibly originated from fecal and terrestrial sources, and the remaining 15% of bacteria came from freshwater and marine sources Pathogens and opportunistic pathogens were also detected, such as *Streptococcus pneumoniae* and *Aspergillus fumigatus* (Cao et al., 2014). In the present study, we report that people could inhale the bacteria or pathogens carried by PM2.5 or PM10 through the respiratory route, as evidenced by the increased diversity of microbiota in pharyngeal swabs, which may lead to an increase in respiratory diseases.

## Materials and Methods

### Study Design

We sampled the pharyngeal mucosa using swabs from the participants on January 16 (before a two-day severe smog episode) and on January 19, 2017 (after the episode), to explore the possible difference in the composition of the microbiota. If the number of bacterial species or the number of a given bacterial species detected in the post-smog sample were statistically significantly higher than that in the pre-smog sample, the increased number was considered to be caused by exposure to the severe smog episode.

We selected the pharyngeal microbiota to study inhalable PM-associated bacteria, which play a significant role in the development of respiratory tract diseases. Vendors in an open-air farmer’s market were selected for this study because they represented a group of people who worked in the same place and were exposed to smog over the same business hours. The outdoor farmer’s market in Liaocheng City of Shangdong Province was selected for this study. The market is approximately 10,000 m^2^, with approximately 300 small vendors. The market is open daily from 6 o’clock a.m. to 12 o’clock a.m. After the market was closed at noon, some of the vendors stayed for additional business hours in the afternoon. Generally, approximately 3,000 customers visited the market each day. Most of them were nearby residents.

The timing of study initiation was carefully calculated using information from the official air quality forecast, which is released to the public every day. Sampling was conducted by scientists from the local Center for Disease Control and Prevention (CDC), who are authorized for such activity. The vendors sampled were fully informed and agreed to participate in the study.

The PM2.5 concentrations in Liaocheng City of Shandong Province in January 2017 fluctuated by approximately 100 μg/m^3^ in a single day. We decided to sample the pre-smog pharyngeal swab on January 16, 2017, when the PM2.5 concentration was reduced from 128 μg/m^3^ to about 80 μg/m^3^ for three consecutive days and was forecasted to increase to 220 μg/m^3^ for the next three consecutive days. The PM2.5 indeed reached a peak of 287 μg/m^3^ on January 18 and maintained a level of 217 μg/m^3^ on January 19, when we sampled the post-smog pharyngeal swab (Supplementary Figure S1). The change in PM10 was consistent with that of PM2.5. The PM10 concentrations were about 150 μg/m^3^ for three consecutive days before the first sampling and were 314 and 416 μg/m^3^ on January 16 and 17, respectively, the two days between first and second samplings.

A total of 83 vendors participated in the study. They were sampled twice, once before and once after the smog event. The vendors included 39 males and 44 females aged 21 to 60 years (Table 1). Relevant information recorded during sampling included working hours (morning only or whole day), whether they wore a mask, smoking, history of antibiotic administration in the past 30 days, and history of clinical respiratory symptoms in the past two weeks (Supplementary Table S1).

**Table 1:** Baseline characteristics of the participants in this study.

Characteristics	Male (*n* = 39)	Female (*n* = 44)
Age (years)	37.9 (9.2)	40.5 (8.9)
Smoking	18 (46%)	3 (7%)
Wore mask	8 (21%)	30 (68%)
Using antibiotics in past 30 days	10 (26%)	9 (20%)
Having respiratory symptoms in past two weeks	6 (15%)	9 (20%)
Working hours		
All day	23 (59%)	26 (59%)
Morning	16 (41%)	18 (41%)
Housing type		
Small bungalow	18 (46%)	24 (55%)
Apartment	21 (54%)	20 (45%)
Accommodations		
Joint rent	11 (28%)	7 (16%)
Live with family	28 (72%)	37 (84%)


The pharyngeal samples were collected from every subject using collection swabs and placed in a collection tube for pharyngeal swabs (Hope Bio-Technology Co., Ltd., Qingdao, China) containing 3 ml of phosphate-buffered saline (PBS). The tubes with pharyngeal specimens were immediately place in an icebox and transported to the laboratory of the local CDC where the DNA extraction was performed.

### High-Throughput *16S rRNA* Sequencing and Phylogenetic Analysis

From the pharyngeal samples, total genomic DNA was extracted using a QIAamp DNA mini-kit (Qiagen, Dusseldorf, Germany). The V3–V4 region of the 16S rRNA gene was amplified by PCR using universal primers targeting most bacteria (F: 5′-CCTAYGGGRBGCASCAG-3′, R: 5′-GGACTACNNGGGTATCTAAT-3′) with a 6-bp barcode unique to each sample. The PCR conditions were 94°C for 4 min; followed by 30 cycles of 94°C for 30 s, 54°C for 30 s, and 72°C for 30 s; and then 72°C for 5 min. The single amplifications were performed in 25 μL reactions with 50 ng of template DNA, which was quantified using a Qubit 2.0 Fluorometer (Thermo Fisher Scientific, Waltham, MA, United States). The expected size of the amplicon amplified by the primers used in this study was 485 bp. The resulting amplicons were purified, quantified, pooled, and sequenced on an Illumina HiSeq 2500 PE-250 platform (Illumina, San Diego, CA, United States) using pair-end sequencing (2 × 250 bp).

Barcodes and sequencing primers were trimmed before the paired end reads were merged using FLASH (V1.2.7^1^), and quality filtering was performed under specific filtering conditions (-q 19) using QIIME (Caporaso et al., 2010). Chimeric and incomplete extension sequences generated in the PCR process were also filtered out using the UCHIME algorithm (UCHIME Algorithm^2^). After processing, sequence analysis was performed using the Uparse software (Uparse v7.0.1001^3^). Sequences with ≥97% similarity were assigned to the same operational taxonomic units (OTUs). Representative sequences for each OTU were screened for further annotation using the RDP-classifier (Version 2.2^4^) against the SILVA_123 database with an 80% confidence level. OTU representative sequences were identified using MUSCLE software (Version 3.8.31), and a phylogenetic tree was built using the FastTree algorithm.

### Statistics

Alpha- and beta-diversity analyses were performed using QIIME and displayed using the R software. Non-parametric Wilcoxon rank-sum tests were used to test the hypothesis. Principal component analysis (PCA), permutational analysis of variance (PERMANOVA), and redundancy analysis (RDA) were performed in the R software (Version 2.15.3). Linear discriminant analysis effect size (LEfSe) was used to detect unique biomarkers by determinations of the relative abundances of the members of the bacterial taxonomies. The differences between alpha diversity indices and the profiles of specific respiratory pathogens on the pre- and post-smog pharynx swabs were tested using paired *t*-tests. Normality of the data was confirmed before the paired *t*-tests were carried out. For all statistical testing of 16S rRNA data, *P*-values were corrected for multiple tests using the Benjamini and Hochberg method.

### Safety

There were no unexpected, new, and/or significant hazards or risks associated with the reported work.

### Ethics Statement

Ethical approval for this study was obtained from the Ethical committee of National Institute for Communicable Disease Control and Prevention Chinese Center for Disease Control and Prevention (ICDC-2018001). All participants provided written informed consent.

## Results

A total of 166 pharyngeal swabs were sampled from 83 vendors, two for each vendor. We obtained a total of 10,585,553 high-quality bacterial 16S rRNA sequences (Supplementary Table S2). The sequences were on average 420.2 ± 2.6 bp in length, ranging from 403 to 425 bp. Each swab yielded an average of 66,362 sequences yielded, varying from 36,251 to 80,311 reads. The 10,585,553 sequences were clustered into 5,975 OTUs with 97% identity, with an average of 533 ± 252 OTUs per swab (Supplementary Table S2). Overall, 47 phyla, 88 classes, 177 orders, 366 families, and 791 genera were annotated for the 5,975 OTUs.

### The Bacterial Profiles

**FIGURE 1:**
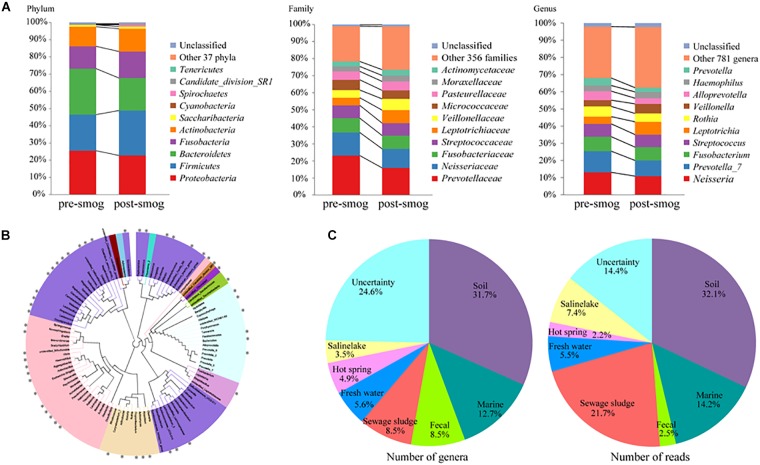
The pharyngeal microbiota composition diversity was affected by smog. **(A)** The pharyngeal microbiota composition diversity at the phylum, family, and genus levels. **(B)** The top 100 most abundant genera between the pre- and post-smog data. The sizes of the nodes correspond to the relative abundance at the corresponding levels in the cohort. **(C)** The origins of 142 new bacterial genera detected only in post-smog samples.

The 47 annotated phyla accounted for 99.33% of the total reads. The top five most abundant phyla accounted for the majority of total reads, at 97.6% and 96.3% for the pre-smog and post-smog swabs, respectively (Figure 1A), and included *Proteobacteria* (24.1% of the reads), *Firmicutes* (23.8%), *Bacteroidetes* (22.6%), *Fusobacteria* (14.1%), and *Actinobacteria* (12.3%). The number of phyla in the post-smog swabs increased from 36 to 46 compared with that of the pre-smog swabs. There were 11 new phyla: SHA-109, *Woesearchaeota*_DHVEG-6, *Caldiserica*, *Chlamydiae*, *Parcubacteria*, MEG, WD272, TA06, *Atribacteria*, *Microgenomates*, and WCHB1-60.

The relative abundance of 38 phyla on the post-smog swabs were increased compared with those of the pre-smog swabs (Supplementary Table S3). Among the five most abundant phyla, the read numbers of *Firmicutes*, *Fusobacteria*, and *Actinobacteria* were increased in the post-smog samples. The increased read numbers for *Firmicutes*, *Fusobacteria*, and *Actinobacteria* were 343,931, 169,013, and 160,158, respectively.

A total of 791 genera were annotated, accounting for 97.82% of total reads. The top ten most abundant genera accounted for 69.0% of total sequences, and included *Prevotella*, *Neisseria*, *Fusobacterium*, *Streptococcus*, *Leptotrichia*, *Rothia*, *Veillonella*, *Alloprevotella*, *Haemophilus*, and *Actinomyces*. The profiles of the top 100 most abundant genera are shown in Figure 1B. The number of genera in the post-smog samples increased from 649 to 767 compared with that in pre-smog swabs. The read numbers of 559 genera were increased in the post-smog swabs (Supplementary Table S4). The top ten increased genera were *Leptotrichia*, *Corynebacterium*, *Veillonella*, *Dolosigranulum*, unidentified_*Chloroplast*, *Moraxella*, *Gemella*, *Actinomyces*, *Granulicatella*, and *Haemophilus*, with more than 20,000 reads for each.

There were 142 new genera detected only in the post-smog swabs (Supplementary Table S5). The origins of those 142 new genera were analyzed according to the published nomenclature and isolation information. Most of them were from soil (31.7%), marine (12.7%), feces (8.5%), sewage sludge (8.5%), freshwater (5.6%), hot springs (4.9%), and saline lakes (3.5%) (Figure 1C). Among the 142 new genera in the post-smog swabs, 35 were detected in PM2.5 and PM10 pollutants during a severe smog event in Beijing in 2013 (Supplementary Table S5; Cao et al., 2014).

### The Variation in Microbiota Between Pre- and Post-smog Pharynx Swabs

All six alpha diversity indices of the post-smog swabs were greater than that of pre-smog swabs. Among them, the indices of richness showed significant differences, including the number of observed species (*P* < 0.001), chao1 (*P* < 0.001), and ACE (*P* < 0.001). Furthermore, the evolutionary distance index and whole-tree phylogenetic diversity showed significant differences (*P* value < 0.05) between the pre- and post-smog data. However, the differences in evenness between the pre-smog and post-smog data were not significant (Supplementary Figure S2 and Supplementary Table S6). Thus, the richness and evolutionary distance of the pharyngeal microbiota were significantly increased by two days of exposure to this smog event.

Compared with levels observed in the pre-smog pharynx samples, the pharyngeal microbiota of the post-smog samples was enriched for the genus *Leptotrichia*, the family *Veillonellaceae*, and the class *Bacilli*, and reduced for the genus *Prevotella_7* and the class *Betaproteobacteria* (*LDA* score > 4.0) (Supplementary Figure S3).

### Factors Influencing the Pharyngeal Microbiota

The principal component analysis (PCA) showed that the composition of the pharyngeal microbiota changed significantly after exposure to high levels of PM2.5, as revealed by separable clusters in the pre- and post-smog samples (Supplementary Figure S4A). This finding was further confirmed by permutational analysis of variance (PERMANOVA) (*P* value = 0.001).

The redundancy analysis (RDA) results (Supplementary Table S7 and Supplementary Figure S4B) showed that the smog event, gender, wearing a mask, and smoking had a significant influence on the structure of the pharyngeal microbiota (*p* < 0.001). No significant effects were observed for working hours, history of using antibiotics, or having respiratory symptoms.

**FIGURE 2:**
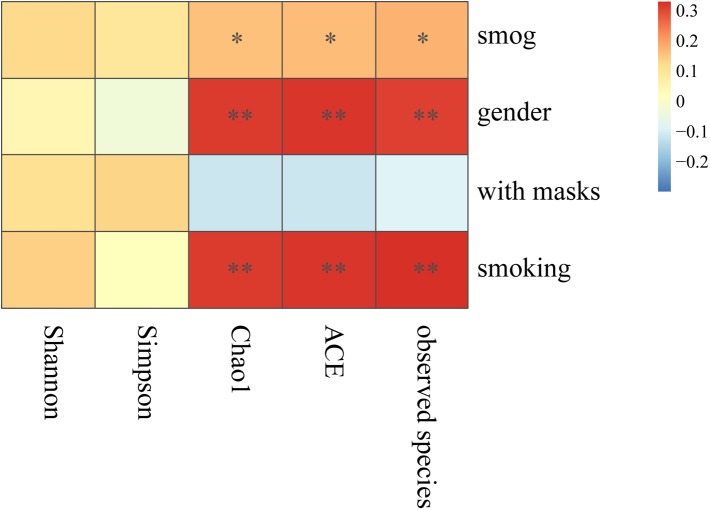
Spearman correlation analysis of influential factors and alpha diversity. ^∗^*p* < 0.05, ^∗∗^*p* < 0.001.

Furthermore, Spearman correlation analysis was carried out to test the correlation between the significant influential factors and alpha diversity (Figure 2). The richness indices (including number of observed species, chao1, and ACE) showed significant positive correlations with the smog event, gender, and smoking. In contrast, wearing a mask was negatively correlated with richness, but the correlation was not significant. Both evenness indices, Shannon and Simpson, correlated positively with the smog event and smoking, but the correlations were not significant.

To further analyze the effect of wearing masks, we analyzed the alpha diversity indexes of the pre- and post-smog pharyngeal microbiota from people wearing and not wearing masks. Among the people wearing masks, no significant difference was observed between alpha diversity indices of the pre- and post-smog data (Supplementary Table S8); however, among those not wearing masks, significant differences were observed of the richness indices (number of observed species, Chao1, ACE) between the pre- and post-smog data. Furthermore, the whole-tree phylogenetic diversity showed significant differences (*P* value = 0.01) among people not wearing masks (Supplementary Table S9). Both gender and smoking were positively correlated with alpha diversity. Notably, the percentage of smokers among males was significantly higher than that in females in this cohort (Supplementary Table S10). Therefore, the data from the 39 male vendors were further analyzed in detail. The RDA analysis showed that smoking had a significant influence on the diversity of the pharyngeal microbiota at the genus level (Supplementary Table S11).

**FIGURE 3:**
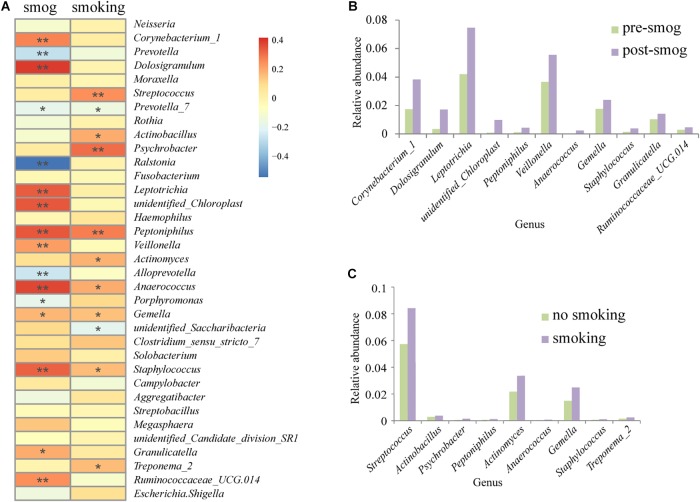
Correlation analysis of smog and smoking with the genera of the pharyngeal microbiota. **(A)** Spearman correlation analysis of influential factors and genera. ^∗^*p* < 0.05, ^∗∗^*p* < 0.001. **(B)** The abundance of eleven genera with positive correlations with smog. **(C)** The abundance of nine genera with positive correlations with smoking.

Based on the Spearman correction analysis, 11 genera correlated positively with the smog event, including *Corynebacterium_1*, *Dolosigranulum*, *Leptotrichia*, *unidentified_Chloroplast*, *Peptoniphilus*, *Veillonella*, *Anaerococcus*, *Gemella*, *Staphylococcus*, *Granulicatella*, and *Ruminococcaceae_UCG.014* (Figure 3A). All of them showed higher abundance in the post-smog data than that in the pre-smog data (Figure 3B), and nine of them were recognized as pathogenic to humans (Supplementary Table S12). The presence of nine genera correlated positively with smoking, including *Streptococcus*, *Actinobacillus*, *Psychrobacter*, *Peptoniphilus*, *Actinomyces*, *Anaerococcus*, *Gemella*, *Staphylococcus*, and *Treponema_2*. All of them showed higher abundance on swabs from people who smoked than on those from non-smokers (Figure 3C), and seven of them were recognized as pathogenic to humans (Supplementary Table S12).

**FIGURE 4:**
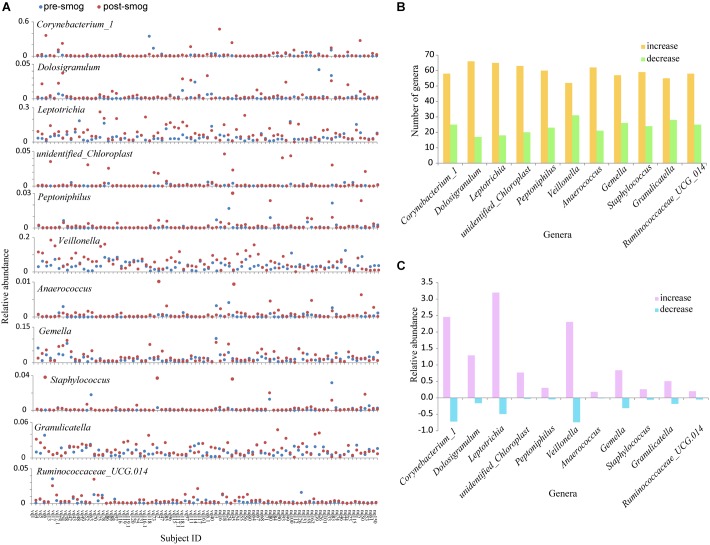
The changes in the relative abundance of the 11 genera showing positive correlations with the smog event. **(A)** The changes in the relative abundance of the 11 genera in each subject. **(B)** The increased and decreased subject numbers for the 11 genera. **(C)** The total increase and decrease in the relative abundances of the 11 genera in the 83 subjects.

The changes in the relative abundance of the 11 genera in each subject were different. The abundance in some subjects increased obviously, however, the increase or decrease in most subjects was not significant (Figure 4A). There were more than 50 subjects who showed increases in the relative abundance of each of the 11 genera, and less than 30 showed decreases (Figure 4B). For the 11 genera, the total increase in their relative abundance in the 83 subjects was significantly larger than their decrease (*P* < 0.05) (Figure 4C).

### The Main Respiratory Pathogen Profile

The profiles of the main recognized respiratory pathogens on the swabs were also analyzed, including *Streptococcus*, *Haemophilus*, *Moraxella, Neisseria*, and *Staphylococcus*. These five genera were detected from both pre- and post-smog samples. The relative abundance of *Streptococcus*, *Haemophilus*, *Moraxella*, and *Staphylococcus* increased in the post-smog swabs. Among them, the relative abundance of *Staphylococcus* increased by up to 300%. Interestingly, the pathogens *Haemophilus influenza* and *Moraxella catarrhalis* were annotated, showing 240% and 150% increases in relative abundance, respectively, compared with that of the pre-smog samples (Supplementary Figure S5). However, these increases were not significant (*P* > 0.05) (Supplementary Table S13). Among the respiratory pathogens, there was a significant reduction in the relative abundance of *Neisseria* between the post- and pre-smog data (*P* < 0.05).

## Discussion

Several studies showed that exposure to PM2.5/PM10 harmed the human respiratory system and caused respiratory diseases (Zanobetti et al., 2003; Xing et al., 2016; Liu et al., 2017). In addition, the pharyngeal microbiota could affect the development of respiratory tract diseases (Teo et al., 2015; Santee et al., 2016; Esposito and Principi, 2018). However, how PM2.5/PM10 exposure affects the human pharyngeal microbiome composition remains poorly understood. In the present study, we reported that exposure to high levels of PM2.5/PM10 critically altered the pharyngeal microbiota composition. The pharynx is the main port of entry for bacterial pathogens and its microbiota can regulate health and disease development, especially the spread of respiratory diseases (Eames et al., 2009; Clifton and Peckham, 2010; HEI Collaborative Working Group on Air Pollution, Poverty, and Health in Ho Chi Minh City et al., 2012).

We hypothesized that high-level PM2.5/PM10 exposure might lead to alterations in the human pharyngeal microbiota composition. Therefore, we compared the profiles of bacterial phylotypes and the abundance of the pharyngeal microbiota sampled pre- and post-smog in a cohort of 83 subjects. All six alpha diversity indices between the pre- and post-smog pharyngeal microbiota were different. Among them, the indices of richness, the evolutionary distance index, and the whole-tree phylogenetic diversity were significantly different, including the number of observed species. PCA analysis showed that the composition of the post-smog pharyngeal microbiota was significantly diversified compared with that of the pre-smog microbiota (Supplementary Figure S3), which was confirmed by permutational analysis of variance. Our results also suggested that wearing a mask could reduce the effect of high-level PM2.5/PM10 exposure on the pharyngeal microbiota, because significant differences were observed for the richness indices and whole-tree phylogenetic diversity between the pre- and post-smog swabs among the people not wearing masks, but not among those wearing masks.

The relative abundance of 38 phyla increased in the post-smog pharyngeal microbiota, such as *Firmicutes*, *Fusobacteria*, and *Actinobacteria*. In addition, 11 new phyla were detected, including *Caldiserica*, *Chlamydiae*, *Parcubacteria*, *Atribacteria*, and *Microgenomates*. At the genus level, the number of phylotypes increased from 649 to 767 in the post-smog pharyngeal microbiota. The relative abundance of 559 genera increased, such as *Leptotrichia*, *Corynebacterium*, *Veillonella*, *Dolosigranulum*, unidentified_*Chloroplast*, *Moraxella*, *Gemella*, *Actinomyces*, *Granulicatella*, and *Haemophilus*. The 142 new genera in post-smog microbiota originated from soil, marine, feces, sewage sludge, freshwater, hot springs, and saline lakes (Figure 1). The main sources of smog are local emissions, ambient dust, and atmospheric circulation. The main sources of ambient dust and atmospheric circulation are the soil and sea, respectively. Therefore, it is reasonable to hypothesize that the 142 new genera in the post-smog microbiota mainly originated from soil and marine sources.

The five major respiratory pathogens were detected in both the pre- and post-smog pharyngeal microbiota. The relative abundance of *Streptococcus*, *Haemophilus*, *Moraxella*, and *Staphylococcus* increased in the post-smog pharyngeal microbiota. Although 16S rDNA sequencing is generally considered to be unable to identify species, the pathogens *H. influenza* and *M. catarrhalis* were annotated in this study. At the species level, the relative abundance of *H. influenza* and *M. catarrhalis* increased by 240 and 150%, respectively. *H. influenza* and *M. catarrhalis* in the nasopharynx are the source of several of the most prevalent causes of morbidity and mortality in humans, such as acute otitis media, pneumonia, meningitis, and bacteremia (Walker et al., 2013). One limitation of this study is that we did not conduct a prospective cohort study; therefore, whether smog exposure caused any diseases is unknown. Furthermore, we only carried out 16S rRNA sequencing without culturing the microorganisms; therefore, the pathogenicity of the identified bacteria was not evaluated.

Interestingly, RDA showed that smoking had a significant effect on the composition of the pharyngeal microbiota (*p* < 0.001) (Supplementary Table S7). Smoking was recorded in 18 of 39 males and only in three of 44 females (Supplementary Table S10); therefore, we extracted data from the 39 male vendors for detailed analysis. Again, a statistically significant effect was found (Supplementary Table S11). The presence of nine bacterial genera was found to be associated with smoking: *Actinomyces*, *Actinobacillus*, *Anaerococcus*, *Peptoniphilus*, *Staphylococcus*, *Streptococcus*, *Gemella, Psychrobacter*, and *Treponema*. Among them, *Streptococcus* and *Staphylococcus* are considered major respiratory pathogens. It was reported that cigarette smoking impaired the host response to *S. pneumoniae* in the nasal mucosa of mice (Shen et al., 2016), and exposure to tobacco smoke increased *S. pneumoniae* and *M. catarrhalis* carriage rates in humans (Greenberg et al., 2006; Bakhshaee et al., 2012). Various effects of smoking on bacterial phylotypes have been reported, such as *Actinomyces* (Rippon and Kathuria, 1984), *Gemella* (Aibar-Arregui et al., 2012), *Actinobacillus* (Tangada et al., 1997), and *Staphylococcus* (Durmaz et al., 2001).

In summary, we reported that exposure to two days of smog altered the composition of the pharyngeal microbiota, which may introduce unexpected health risks, especially respiratory infections. The concentrations of PM2.5/PM10 in Liaocheng City of Shandong Province in 2017 were far in excess of the level recommended by the World Health Organization’s guidelines on air quality and this may be the case due to chronic exposure to levels above those recommended by the WHO. The effect of high concentrations of PM2.5/PM10 on the composition of pharyngeal microbiota might be more severe than that represented by the data reported in the present study.

## Author Contributions

JX conceived the study. TQ, FZ, and HZ performed the literature search and wrote the protocols. FZ, YD, SL, FW, LC, and XX recruited the vendors and collected the specimens. TQ, HZ, YD, HR, and SL performed the laboratory analyses. TQ, HZ, and AJ analyzed the data. TQ, HZ, AJ, YW, JZ, and JX interpreted the data. All authors contributed to the writing and editing of the report and approved the final version.

## Conflict of Interest Statement

The authors declare that the research was conducted in the absence of any commercial or financial relationships that could be construed as a potential conflict of interest.

## Footnotes

1 http://ccb.jhu.edu/software/FLASH/

2 http://www.drive5.com/usearch/manual/uchime_algo.html

3 http://drive5.com/uparse/

4 http://sourceforge.net/projects/rdp-classifier/
