# Supplementary material for: High-Level PM2.5/PM10 Exposure Is Associated With Alterations in the Human Pharyngeal Microbiota Composition
Source: Front Microbiol. 2019 Jan 28;10:54. doi: 10.3389/fmicb.2019.00054 (PMC6379047; doi:10.3389/fmicb.2019.00054)
Supplement: Supplementary file 1 [file Data_Sheet_1.PDF]

Supplementary material

High-level PM2.5 exposure affects human pharyngeal microbiome composition

Tian Qin, Furong Zhang, Haijian Zhou, Hongyu Ren, Yinju Du, Shengnan Liang, Fei Wang, Lihong Cheng, Xuguang Xie, Aoming Jin, Yangfeng Wu, Jinxing Zhao, Jianguo Xu

Table of Contents

Figure S1--page 2

Figure S2--page 3

Figure S3--page 3

Figure S4--page 4

Figure S5--page 5

Table S1---page 6

Table S2---page 9

Table S3--page 19

Table S4--page 22

Table S5--page 56

Table S6--page 60

Table S7--page 61

Table S8--page 62

Table S9--page 63

Table S10--page 64

Table S11--page 65

Table S12--page 66

Table S13--page 67

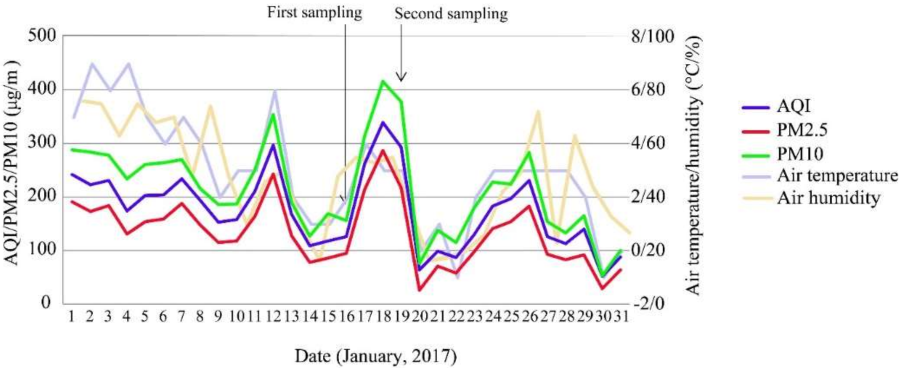

Figure S1. The air quality index, PM2.5, PM10, air temperature and humidity of the days before and between the sampling days. The air temperature and humidity were measured at 10 a.m. when the samples were collected.

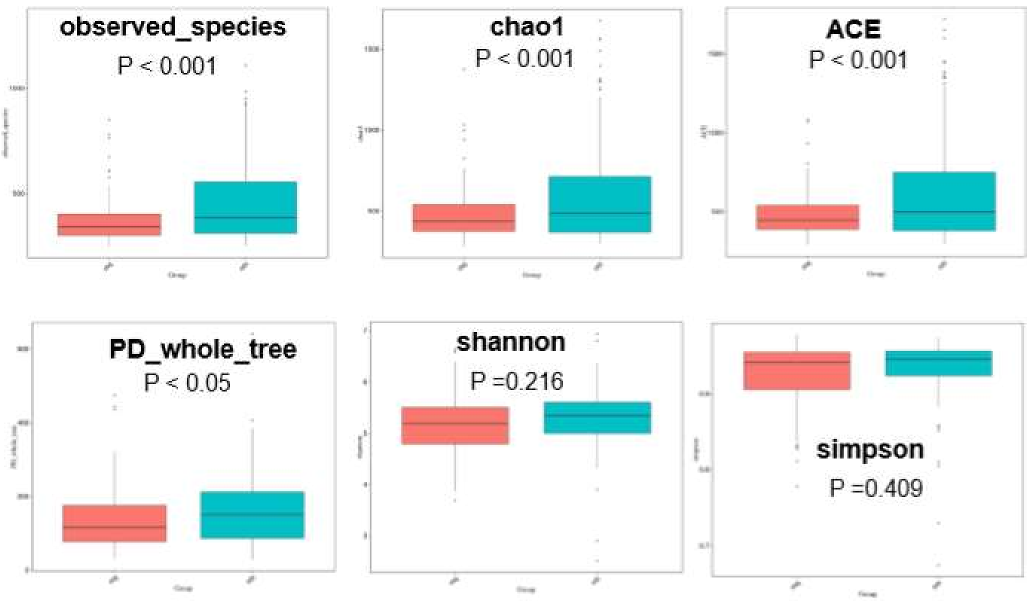

Figure S2. The alpha diversity analysis of microbiota of pre- and post-smog nasopharynx swabs of vendors at open air famer market. Box plots of alpha diversity were generated with rarefication to 63,768 reads per sample. The paired t-test P-values were calculated using SPSS software.

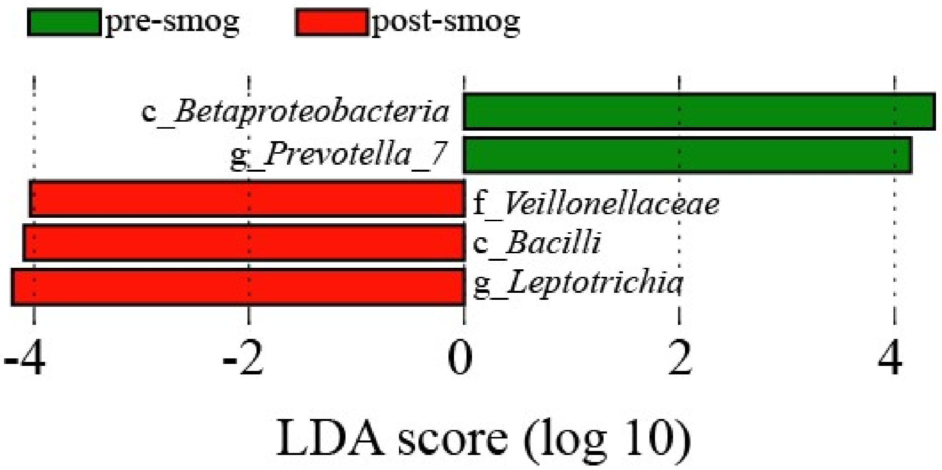

Figure S3. Histogram of unique biomarker bacteria in each group as analyzed by linear discriminant analysis effect size (LEfSe). The LDA effect size (> 4.0-fold) was used to detect unique biomarkers.

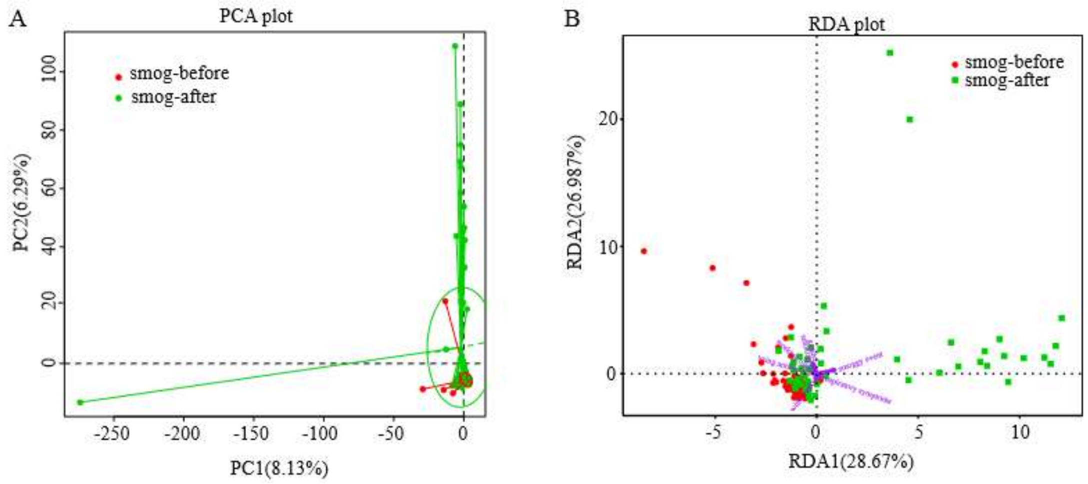

Figure S4. Component and structure of microbiota of pre- and post-smog nasopharynx swabs (PCA+RDA). (A) Principal component analysis; (PCA) plot of microbiota of before smog and after smog exposure; (B) Redundancy analysis (RDA) of the correlation between influential factors and the structure of pharyngeal microbiota.

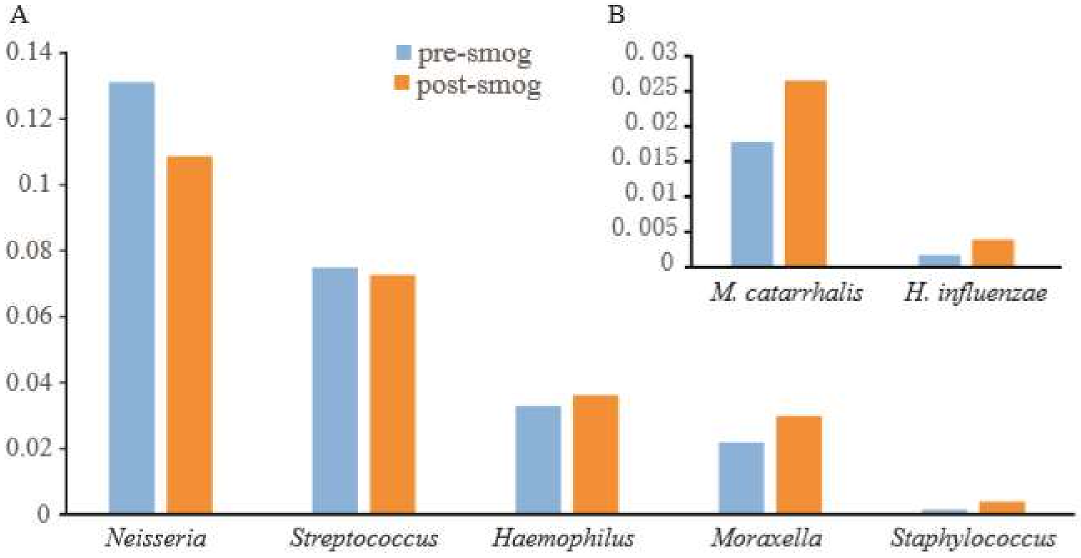

Figure S5. Relative abundance of five genera (A) and two species (B) of respiratory pathogens in pre- and post-smog nasopharynx swabs.

Table S1. The detailed information of the 83 subjects sampled in this study

Subject ID	Age (year-old)	Gender	Working hours (morning/ all day)	With masks (yes or no)	Smoking (yes or no)	History of administration of antibiotics in past 30 days	History with respiratory symptoms in past two weeks
vq1	26	Female	Morning	No	No	Yes	No
vq4	52	Female	All day	No	No	No	No
vq5	40	Female	All day	Yes	No	Yes	No
vq6	43	Female	All day	No	No	No	No
vq6.1	36	Female	All day	Yes	No	No	No
vq7	47	Female	All day	No	No	No	No
vq13	47	Female	Morning	Yes	No	No	No
vq17	44	Female	Morning	Yes	No	No	No
vq17.1	30	Female	Morning	Yes	Yes	Yes	Yes
vq28	47	Female	Morning	Yes	No	No	Yes
vq38	42	Female	Morning	Yes	No	No	No
vq40	46	Female	Morning	Yes	Yes	No	No
vq42	28	Female	All day	Yes	No	No	No
vq43	39	Female	Morning	Yes	No	No	No
vq47	38	Female	All day	No	No	No	No
vq48	48	Female	Morning	No	No	Yes	No
vq49	47	Female	All day	Yes	No	No	No
vq52	42	Female	All day	Yes	No	No	Yes
vq63	43	Female	All day	Yes	No	No	No
vq70	60	Female	Morning	Yes	No	No	No
vq73	31	Female	All day	No	No	Yes	Yes
vq75	50	Female	All day	No	No	No	No
vq78	49	Female	All day	No	No	No	No
vq85	35	Female	All day	No	No	No	Yes
vq86	46	Female	Morning	Yes	No	Yes	No
vq88	28	Female	Morning	Yes	No	No	No
vq92	37	Female	All day	Yes	No	Yes	No
vq96	54	Female	All day	Yes	No	No	No
vq97	40	Female	Morning	Yes	No	Yes	Yes
vq111	40	Female	All day	No	No	No	No
vq114	60	Female	Morning	Yes	No	No	No

vq115	33	Female	All day	No	No	No	No
vq116	50	Female	Morning	Yes	Yes	No	No
vq116.1	33	Female	Morning	Yes	No	No	No
vq117	27	Female	All day	Yes	No	No	No
vq117.1	28	Female	All day	Yes	No	Yes	No
vq118	41	Female	All day	Yes	No	No	Yes
vq118.1	38	Female	All day	Yes	No	No	No
vq119	31	Female	All day	No	No	No	No
vq119.1	54	Female	All day	No	No	No	No
vq120	34	Female	Morning	Yes	No	No	Yes
vq125	31	Female	Morning	Yes	No	No	Yes
vq131	39	Female	All day	Yes	No	No	No
vq161	30	Female	All day	Yes	No	No	No
nq2	26	Male	All day	No	Yes	No	No
nq3	53	Male	All day	No	No	No	No
nq16	39	Male	All day	Yes	No	No	No
nq18	45	Male	All day	Yes	Yes	No	No
nq23	22	Male	All day	No	No	No	No
nq24	42	Male	All day	No	No	No	No
nq26	40	Male	All day	No	No	Yes	No
nq33	24	Male	Morning	Yes	No	Yes	Yes
nq39	47	Male	All day	No	No	No	No
nq44	38	Male	Morning	No	Yes	No	No
nq45	31	Male	Morning	No	Yes	No	Yes
nq46	21	Male	Morning	Yes	No	No	No
nq50	33	Male	All day	No	Yes	Yes	No
nq53	46	Male	All day	No	No	No	No
nq54	28	Male	All day	No	Yes	No	Yes
nq55	44	Male	Morning	No	No	No	Yes
nq56	24	Male	Morning	Yes	No	Yes	No
nq60	44	Male	Morning	No	No	No	No
nq61	39	Male	All day	No	Yes	No	No
nq64	44	Male	Morning	No	Yes	No	No
nq67	44	Male	All day	No	No	No	No
nq68	50	Male	All day	Yes	No	No	No
nq71	40	Male	All day	No	Yes	No	No
nq74	44	Male	All day	No	Yes	No	Yes
nq80	57	Male	Morning	Yes	No	Yes	No

nq83	51	Male	Morning	Yes	No	No	No
nq84	40	Male	All day	No	No	No	No
nq90	43	Male	Morning	No	Yes	No	No
nq93	29	Male	All day	No	Yes	Yes	Yes
nq99	44	Male	All day	No	Yes	No	No
nq100	31	Male	All day	No	No	Yes	No
nq101	33	Male	All day	No	Yes	No	No
nq112	38	Male	Morning	No	Yes	Yes	No
nq115	30	Male	Morning	No	Yes	No	No
nq126	35	Male	Morning	No	Yes	No	No
nq150	45	Male	Morning	No	Yes	Yes	No
nq151	39	Male	All day	No	No	No	No
nq155	26	Male	Morning	No	No	No	No
nq162	35	Male	All day	No	No	Yes	No

Table S2. The summary of analyzed sequences information

Sample	Q20 (%)	Q30 (%)	GC%	Raw reads	Combined	Qualified	Extract effective	Total _tag	Length	Taxon _Tag	Unclassi fied_Tag	Unique _Tag	OUT _num
vq1	98.42	96.82	51.93	94,987	86,715	72,371	66.54	63,200	424	62117	27	1056	481
vq4	98.53	97.13	50.97	87,824	80,477	70,457	74.57	65,489	420	64693	2	794	295
vq5	98.46	96.94	51.8	90,637	82,393	70,334	75.5	68,429	423	68034	46	349	381
vq6	98.52	97.11	50.15	81,126	73,754	64,532	76.28	61,885	416	61506	10	369	443
vq6.1	97.48	95.21	51.21	83,570	80,397	56,272	58.52	48,901	418	47722	1	1178	374
vq7	97.23	94.75	51.77	78,937	74,811	47,104	57.23	45,172	424	44916	2	254	316
vq13	98.48	96.98	51.9	85,117	76,521	66,588	73.67	62,704	419	62154	6	544	450
vq17	98.48	97.01	51.64	75,787	66,475	57,534	73.8	55,929	417	55085	227	617	867
vq17.1	98.45	96.95	52.03	98,410	89,237	76,602	76.22	75,008	421	74575	72	361	659
vq28	98.51	97.06	51.26	70,319	64,257	55,543	76.66	53,908	421	53547	30	331	463
vq38	98.51	97.06	51.55	96,737	88,872	76,479	75.46	72,996	423	72530	16	450	379
vq40	97.31	94.87	51.86	81,115	78,173	50,422	59.97	48,647	419	47624	86	937	722
vq42	98.54	97.14	50.89	59,869	53,902	47,125	76.28	45,667	417	45377	5	285	327
vq43	98.46	96.98	51.93	87,170	78,786	67,648	73.3	63,894	420	63505	31	358	466

vq47	97.3	94.87	51.65	79,670	76,560	51,007	59.95	47,759	419	47186	0	573	299
vq48	98.47	96.98	52.19	73,468	66,514	57,036	75.56	55,512	422	55155	38	319	493
vq49	98.52	97.08	52.66	86,177	80,159	69,023	78.39	67,555	424	67290	5	260	312
vq52	98.52	97.08	52.66	66,945	60,974	52,350	75.73	50,695	421	50320	96	279	527
vq63	98.48	96.96	51.75	81,794	73,229	63,537	71.55	58,520	421	58049	5	466	323
vq70	98.45	96.95	51.58	79,318	71,856	61,908	75.09	59,558	421	59220	4	334	372
vq73	97.41	95.11	51.52	83,233	79,653	54,455	58.26	48,491	420	47808	9	674	364
vq75	98.48	97.01	51.34	80,784	72,477	62,966	74.32	60,035	419	59687	23	325	395
vq78	98.5	97.03	53.96	87,547	80,572	68,757	76.94	67,359	420	66801	18	540	386
vq85	97.25	94.79	51.76	77,583	73,628	48,063	59.35	46,044	421	45708	26	310	377
vq86	98.5	97.02	53.17	86,549	78,604	67,624	73.26	63,410	421	63118	14	278	394
vq88	98.45	96.9	52.71	80,892	72,414	62,307	74.63	60,372	421	60031	10	331	463
vq92	98.5	97.07	51.37	96,672	86,491	74,581	75.66	73,145	421	72828	51	266	493
vq96	97.34	94.99	51.05	91,137	85,861	56,120	57.98	52,844	423	52418	1	425	306
vq97	97.41	95.09	51.95	85,791	82,644	55,272	60.44	51,848	424	51517	19	312	396
vq111	97.29	95.01	51.57	86,743	82,636	54,676	61.38	53,241	424	53064	31	146	408
vq114	97.39	94.99	51.97	96,575	90,896	59,180	59.46	57,425	421	56920	3	502	404

vq115	97.29	94.86	51.88	85,590	81,626	52,898	57.92	49,576	422	49025	0	551	325
vq116	98.57	97.2	51.07	82,321	75,060	65,158	76.57	63,037	422	62779	22	236	398
vq116.1	97.23	94.64	54	75,142	70,882	47,879	60.96	45,803	417	45591	9	203	364
vq117	97.4	95.07	51.53	89,267	84,734	58,198	57.54	51,360	420	49198	0	2162	323
vq117.1	97.32	94.99	51.31	89,221	86,003	57,159	62.15	55,447	421	55110	32	305	543
vq118	97.25	94.75	52.37	83,066	80,472	52,758	61.39	50,994	420	50700	10	284	391
vq118.1	97.33	94.95	51.03	78,515	75,492	49,580	58.62	46,025	421	45605	6	414	382
vq119	98.49	97.06	51.66	95,672	86,935	74,375	74.5	71,271	422	70822	12	437	456
vq119.1	98.53	97.12	51.44	95,855	87,050	75,412	74.91	71,805	422	71508	18	279	339
vq120	98.5	97.07	51.69	90,206	81,581	70,457	75.47	68,079	420	67588	63	428	561
vq125	98.53	97.14	51.17	93,482	84,871	74,132	76.62	71,629	419	71256	10	363	353
vq131	97.31	94.84	53.74	86,210	81,894	55,555	54.59	47,062	417	45881	1	1180	322
vq161	97.49	95.24	50.93	60,625	57,485	39,817	59.8	36,251	418	35625	3	623	440
nq2	98.23	96.57	51.46	103,342	99,696	80,305	75.1	77,606	421	77293	3	310	484
nq3	98.6	97.27	51.52	80,353	73,831	64,768	77.52	62,293	420	61957	11	325	375
nq16	98.45	96.94	51.41	60,737	53,913	46,684	73.52	44,654	419	44226	149	279	549
nq18	98.45	96.92	52.55	82,512	72,546	61,639	71.75	59,206	422	58599	306	301	707

nq23	98.18	96.47	51.84	95,516	91,329	74,296	74.5	71,160	420	70636	23	501	706
nq24	98.48	97.04	52.69	92,068	84,549	71,716	75.69	69,687	425	69416	8	263	398
nq26	98.2	96.58	51.1	98,871	92,941	75,558	73.34	72,512	423	72230	12	270	439
nq33	98.2	96.5	52.16	98,114	93,969	76,759	74.68	73,275	420	72687	58	530	701
nq39	98.14	96.52	50.96	91,584	87,078	69,883	73.88	67,660	424	67394	22	244	465
nq44	98.21	96.52	51.59	104,249	98,551	79,132	73.74	76,877	420	76456	6	415	615
nq45	98.56	97.19	50.34	96,747	87,711	76,699	77.06	74,555	419	74132	9	414	423
nq46	98.19	96.5	52.01	88,389	84,831	68,949	73.62	65,071	421	64889	5	177	472
nq50	98.24	96.61	51.96	94,605	89,500	73,391	74.57	70,550	419	70047	1	502	531
nq53	98.19	96.52	52.07	98,552	94,622	76,167	73.31	72,245	422	72007	16	222	421
nq54	98.52	97.09	50.56	57,143	51,819	45,320	76.48	43,702	418	43392	1	309	345
nq55	98.49	97.06	50.92	93,828	83,847	72,490	74.76	70,150	422	69572	5	573	332
nq56	98.5	97.04	51.52	80,078	72,465	62,881	76.17	60,994	421	60518	10	466	339
nq60	98.48	96.97	51.82	90,411	82,592	70,974	71.44	64,590	424	63984	7	599	331
nq61	98.18	96.47	51.98	107,638	101,567	81,951	74.61	80,311	423	79969	11	331	543
nq64	98.49	97.01	51.98	98,487	90,296	77,423	75.72	74,571	424	74000	123	448	472
nq67	98.56	97.2	51.2	85,717	78,123	68,106	77.72	66,618	419	66288	9	321	358

nq68	98.47	97	51.24	64,004	58,607	50,516	77.23	49,432	422	49113	33	286	415
nq71	98.5	97.01	51.6	84,493	64,198	53,027	55.86	47,195	420	45494	553	1148	996
nq74	98.27	96.69	50.99	102,993	99,168	81,712	76.59	78,880	421	78612	2	266	416
nq80	98.43	96.9	52.63	94,032	86,570	73,395	76.51	71,944	424	71669	4	271	442
nq83	98.18	96.48	51.4	91,201	87,364	70,375	73.53	67,062	421	66581	5	476	580
nq84	98.54	97.15	50.31	93,002	85,014	74,321	78.11	72,640	418	72191	0	449	358
nq90	98.4	96.81	53.26	89,128	81,090	68,258	75.17	67,001	423	66463	44	494	568
nq93	98.43	96.86	53.64	95,568	86,976	74,388	76.13	72,755	420	72347	27	381	524
nq99	98.5	97.03	52.05	73,155	66,755	57,445	76.4	55,890	419	55598	23	269	465
nq100	98.49	96.99	52.72	81,222	74,229	63,891	74.52	60,530	422	60190	41	299	488
nq101	98.23	96.6	51.11	104,901	98,817	81,046	73.09	76,671	421	76119	0	552	429
nq112	98.53	97.12	51.54	91,620	83,843	72,341	76	69,635	423	69312	13	310	376
nq115	98.22	96.55	51.88	94,080	88,745	72,672	75.75	71,267	419	71032	13	222	471
nq126	98.47	97	51.43	70,195	61,742	53,392	73.7	51,734	418	51151	250	333	614
nq150	98.2	96.53	51.3	101,896	96,659	79,026	76.15	77,591	420	77293	38	260	569
nq151	98.49	97.03	51.57	88,919	81,467	69,807	76.52	68,040	422	67777	39	224	448
nq155	98.5	97.05	51.12	89,804	79,067	68,417	73.9	66,362	418	65439	303	620	918

nq162	98.49	97.01	51.03	83,017	75,764	65,623	71.3	59,192	420	58794	44	354	444
vh1	98.52	97.12	50.62	93,209	85,269	73,971	72.33	67,414	419	67213	20	181	371
vh4	98.46	96.94	51.27	82,017	74,727	64,578	76.21	62,506	420	62250	3	253	318
vh5	98.5	97.01	52.92	97,799	86,301	75,476	75.66	73,993	418	73479	212	302	552
vh6	98.21	96.45	52.22	85,281	74,493	69,050	78.36	66,829	403	66291	59	479	456
vh6.1	97.35	94.96	51.61	75,580	72,593	46,433	57.83	43,706	421	43475	2	229	310
vh7	97.3	94.87	51.82	82,621	79,486	50,445	58.65	48,454	424	48220	11	223	341
vh13	98.49	97.05	51.36	90,333	82,372	71,334	76.5	69,109	419	68917	5	187	341
vh17	98.5	97.05	51.87	95,404	83,721	72,504	75.08	71,631	417	71177	234	220	636
vh17.1	98.49	97.03	52.25	92,320	83,479	72,163	76.9	70,992	419	70610	65	317	668
vh28	98.53	97.07	51.45	98,990	90,942	78,628	77.93	77,138	421	76714	102	322	504
vh38	98.52	97.07	51.82	85,345	78,292	67,051	75.08	64,080	422	63736	35	309	415
vh40	97.16	94.63	52.1	80,209	77,573	48,199	57.52	46,135	422	45687	162	286	499
vh42	98.54	97.14	50.92	83,956	76,201	66,573	77.46	65,032	417	64738	46	248	387
vh43	98.55	97.17	51.26	81,133	74,909	64,702	76.56	62,119	421	61920	13	186	352
vh47	97.38	95	51.97	90,292	85,320	56,513	60.97	55,050	418	54720	0	330	294
vh48	98.47	96.96	52.21	90,149	81,412	69,999	76.43	68,900	419	68520	75	305	464

vh49	98.47	96.99	52.09	86,925	79,013	68,187	76.78	66,741	419	66180	148	413	569
vh52	98.51	97.06	52.01	87,963	81,398	70,122	77.93	68,550	422	68293	17	240	447
vh63	98.56	97.13	51.76	94,531	85,597	74,846	75.9	71,753	419	71454	7	292	351
vh70	98.46	96.97	51.52	95,740	87,287	75,599	76.56	73,295	418	72932	3	360	394
vh73	97.36	95.04	50.72	81,358	76,539	53,243	62.5	50,846	416	50138	203	505	638
vh75	98.54	97.13	51.76	92,450	84,523	73,233	76.52	70,740	420	70526	15	199	381
vh78	98.5	97.03	53.13	82,281	75,769	65,003	76.79	63,186	421	62047	79	1060	1198
vh85	97.39	95.05	51.51	93,505	88,815	59,424	60.81	56,856	419	56558	1	297	318
vh86	98.5	97.06	51.78	87,865	81,441	69,791	75.85	66,647	425	65828	58	761	1032
vh88	98.53	97.12	52.45	96,173	87,961	76,864	77.85	74,869	418	73919	56	894	1308
vh92	98.52	97.15	51.7	91,812	84,922	73,365	78.16	71,761	424	70736	102	923	1165
vh96	97.47	95.23	51.02	86,375	81,837	56,469	63.78	55,088	417	54907	0	181	407
vh97	97.29	94.86	52.21	80,014	75,656	50,407	58.22	46,583	421	46457	7	119	352
vh111	97.29	94.91	51.12	90,347	86,518	58,220	60.65	54,799	419	54431	2	366	289
vh114	97.27	94.85	51.84	88,957	84,885	53,831	58.68	52,198	418	51957	4	237	366
vh115	97.44	95.11	51.4	89,187	86,206	57,914	62.53	55,773	419	55476	0	297	334
vh116	98.52	97.04	52.32	95,879	87,999	76,102	76.41	73,259	421	72065	27	1167	1259

vh116.1	97.23	94.74	52.07	93,099	88,530	54,102	56.05	52,184	424	51903	80	201	373
vh117	97.36	95.05	50.97	80,798	77,960	54,153	64.79	52,350	416	52114	4	232	379
vh117.1	97.24	94.75	52.26	77,031	74,618	47,038	58.52	45,076	421	44874	7	195	318
vh118	97.27	94.82	52.14	92,647	89,148	58,210	61.94	57,382	422	57242	12	128	323
vh118.1	97.41	95.12	50.93	92,876	89,295	62,462	65.7	61,023	418	60853	6	164	340
vh119	98.52	97.08	52.63	95,737	88,074	76,177	75.1	71,903	421	70704	20	1179	1164
vh119.1	98.5	97.03	52.28	90,882	83,128	72,070	75.43	68,549	422	67501	18	1030	1050
vh120	98.52	97.08	52.06	83,951	76,949	66,698	75.83	63,660	420	62276	56	1328	1365
vh125	98.52	97.07	51.91	90,815	82,258	71,446	75.09	68,192	421	67218	11	963	817
vh131	97.41	95.13	52.06	79,756	76,229	55,774	68.15	54,356	413	54117	2	237	364
vh161	97.27	94.97	51.36	82,353	79,216	52,272	60.92	50,167	423	49890	39	238	318
nh2	98.23	96.57	52.67	85,420	81,325	67,009	76.35	65,222	418	64651	273	298	539
nh3	98.54	97.15	52.92	92,703	84,941	74,119	78.67	72,934	418	72766	44	124	435
nh16	98.47	96.98	51.68	96,810	87,671	76,470	77.81	75,330	416	75089	35	206	418
nh18	98.48	96.98	51.89	95,107	87,968	75,619	75.93	72,217	422	71866	37	314	461
nh23	98.24	96.59	51.15	101,327	95,792	78,462	75.26	76,260	422	76007	31	222	589
nh24	98.58	97.24	51.66	96,073	87,037	77,082	78.69	75,597	412	75187	18	392	527

nh26	98.27	96.66	50.93	96,313	91,369	75,725	75.96	73,160	419	72772	2	386	412
nh33	98.21	96.52	52.47	84,837	81,472	67,730	77.21	65,505	416	64509	21	975	854
nh39	98.19	96.56	51.56	94,427	90,322	72,293	74	69,880	424	69635	19	226	424
nh44	98.18	96.47	52.05	93,280	87,827	68,876	72.11	67,267	423	67082	16	169	459
nh45	98.52	97.08	51.69	98,014	86,604	74,888	72.59	71,150	419	70412	299	439	722
nh46	98.24	96.62	50.89	105,198	99,819	82,340	68.95	72,538	421	71854	0	684	416
nh50	98.12	96.33	52.22	103,725	99,847	78,180	73.72	76,467	424	76230	38	199	420
nh53	98.19	96.5	53.9	101,825	97,608	79,953	73.72	75,064	418	74616	1	447	404
nh54	98.5	97.04	51	83,040	75,652	65,911	77.93	64,711	420	64322	83	306	443
nh55	98.54	97.14	51.51	83,600	75,944	65,985	76.36	63,835	418	63494	5	336	419
nh56	98.56	97.17	51.95	99,744	91,393	79,488	76.59	76,392	421	76128	34	230	447
nh60	98.53	97.11	52.3	99,184	91,709	79,470	75.91	75,295	421	75010	10	275	368
nh61	98.17	96.41	53.37	98,989	93,137	75,138	72.73	71,993	421	71642	6	345	500
nh64	98.52	97.08	51.52	89,366	81,452	70,150	76.11	68,019	421	67570	34	415	459
nh67	98.53	97.08	51.65	99,760	89,967	76,848	75.41	75,232	422	74807	115	310	647
nh68	98.74	97.52	50.88	87,784	78,470	69,299	75.26	66,067	420	65189	20	858	662
nh71	98.52	97.08	51.86	98,709	88,542	76,952	76.12	75,137	416	74681	76	380	670

nh74	98.21	96.57	51.21	89,155	86,064	70,565	76.24	67,973	420	67660	21	292	532
nh80	98.56	97.15	52.94	80,409	74,105	64,644	77.3	62,157	420	61318	11	828	1174
nh83	98.2	96.51	51.5	92,997	87,970	71,950	74.34	69,130	420	68869	20	241	557
nh84	98.52	97.09	52.67	87,876	80,969	70,505	77.65	68,235	420	67402	67	766	1134
nh90	98.52	97.09	52.6	89,521	81,561	71,105	75.47	67,559	420	66515	37	1007	964
nh93	98.45	96.9	52.63	90,396	82,297	70,743	75.99	68,691	422	67723	48	920	1020
nh99	98.65	97.36	51.04	82,139	70,924	62,756	72.37	59,440	417	58886	34	520	613
nh100	98.59	97.21	50.97	87,985	71,684	61,666	69.14	60,832	424	59928	196	708	671
nh101	98.2	96.54	51.59	104,308	98,448	80,715	73.98	77,169	419	76890	32	247	635
nh112	98.54	97.13	52.48	92,336	85,457	73,967	76.04	70,215	421	68706	33	1476	1272
nh115	98.19	96.49	51.7	91,275	86,743	70,832	74.44	67,949	419	67528	1	420	591
nh126	98.51	97.04	52.7	91,096	84,304	72,696	76.93	70,076	422	68910	15	1151	1116
nh150	98.13	96.31	53.53	88,273	82,981	67,630	72.41	63,917	420	63513	3	401	536
nh151	98.5	97.03	52.4	97,154	86,447	74,933	74.08	71,970	420	70675	169	1126	1178
nh155	98.62	97.26	51.04	94,613	75,036	66,134	66.89	63,289	417	61262	355	1672	1550
nh162	98.7	97.43	50.81	95,300	77,775	68,279	67.91	64,716	420	64309	68	339	656

Table S3. The information of 38 phyla of post-smog swabs was increased as compared with that of smog-before swabs

Taxonomy	Reads number in pre-smog swabs	Proportion of total of pre-smog swabs	Reads number in post-smog swabs	Proportion of total of post-smog swabs	Total	Proportion of total	The value of changes after the exposure of smog
Firmicutes	1077554	21.13%	1421485	26.27%	2499039	23.7786%	343931
Fusobacteria	657446	12.89%	826459	15.28%	1483905	14.1195%	169013
Actinobacteria	567943	11.14%	728101	13.46%	1296044	12.3320%	160158
Saccharibacteria	49458	0.97%	62813	1.16%	112271	1.0683%	13355
Candidate_division_SR1	9599	0.19%	11145	0.21%	20744	0.1974%	1546
Cyanobacteria	4597	0.09%	52861	0.98%	57458	0.5467%	48264
Tenericutes	2621	0.05%	2945	0.05%	5566	0.0530%	324
Acidobacteria	1799	0.04%	2941	0.05%	4740	0.0451%	1142
Chloroflexi	800	0.02%	975	0.02%	1775	0.0169%	175
Gracilibacteria	402	0.01%	407	0.01%	809	0.0077%	5
Gemmatimonadetes	270	0.01%	2208	0.04%	2478	0.0236%	1938

Deinococcus-Thermus	167	0.00%	168	0.00%	335	0.0032%	1
Nitrospirae	128	0.00%	458	0.01%	586	0.0056%	330
Thermomicrobia	126	0.00%	435	0.01%	561	0.0053%	309
Verrucomicrobia	60	0.00%	307	0.01%	367	0.0035%	247
Elusimicrobia	49	0.00%	67	0.00%	116	0.0011%	18
Latescibacteria	35	0.00%	55	0.00%	90	0.0009%	20
Chlorobi	24	0.00%	2443	0.05%	2467	0.0235%	2419
JL-ETNP-Z39	8	0.00%	106	0.00%	114	0.0011%	98
Thermotogae	7	0.00%	10	0.00%	17	0.0002%	3
Planctomycetes	6	0.00%	29	0.00%	35	0.0003%	23
Armatimonadetes	4	0.00%	15	0.00%	19	0.0002%	11
Aminicenantes	3	0.00%	15	0.00%	18	0.0002%	12
Deferribacteres	3	0.00%	10	0.00%	13	0.0001%	7
TM6	2	0.00%	747	0.01%	749	0.0071%	745
Candidate_division_WS6	2	0.00%	8	0.00%	10	0.0001%	6
Hydrogenedentes	2	0.00%	3	0.00%	5	0.0000%	1
SHA-109	0	0.00%	93	0.00%	93	0.0009%	93

Woesearchaeota_.DHVEG-6.	0	0.00%	36	0.00%	36	0.0003%	36
Caldiserica	0	0.00%	32	0.00%	32	0.0003%	32
Chlamydiae	0	0.00%	27	0.00%	27	0.0003%	27
Parcubacteria	0	0.00%	17	0.00%	17	0.0002%	17
.MEG.	0	0.00%	11	0.00%	11	0.0001%	11
WD272	0	0.00%	9	0.00%	9	0.0001%	9
TA06	0	0.00%	9	0.00%	9	0.0001%	9
Atribacteria	0	0.00%	8	0.00%	8	0.0001%	8
Microgenomates	0	0.00%	4	0.00%	4	0.0000%	4
WCHB1-60	0	0.00%	2	0.00%	2	0.0000%	2

Table S4. The information of 559 genera of post-smog swabs was increased as compared with that of smog-before swabs

Taxonomy (genus level)	Reads in pre-smog swabs	Proportion of total of pre-smog swabs	Reads in post-smog swabs	Proportion of total of post-smog swabs	Total	Proportion of total	The value of changes after the exposure of smog
Leptotrichia	211620	4.1499%	400967	7.4113%	612587	5.8288%	189347
Corynebacterium	81384	1.5960%	216643	4.0043%	298027	2.8358%	135259
Veillonella	191665	3.7586%	301948	5.5811%	493613	4.6968%	110283
Dolosigranulum	17300	0.3393%	93585	1.7298%	110885	1.0551%	76285
unidentified_Chloroplast	4438	0.0870%	51401	0.9501%	55839	0.5313%	46963
Moraxella	109310	2.1436%	154034	2.8471%	263344	2.5057%	44724
Gemella	90047	1.7658%	129905	2.4011%	219952	2.0929%	39858
Actinomyces	152871	2.9978%	188705	3.4879%	341576	3.2501%	35834
Granulicatella	53309	1.0454%	76300	1.4103%	129609	1.2332%	22991
Haemophilus	172922	3.3910%	194999	3.6043%	367921	3.5008%	22077
Peptoniphilus	6397	0.1254%	25600	0.4732%	31997	0.3045%	19203
Megasphaera	18514	0.3631%	33788	0.6245%	52302	0.4977%	15274

Staphylococcus	7412	0.1454%	22168	0.4097%	29580	0.2815%	14756
Atopobium	22268	0.4367%	36261	0.6702%	58529	0.5569%	13993
unidentified_Saccharibacteria	44578	0.8742%	58469	1.0807%	103047	0.9805%	13891
Streptococcus	384856	7.5471%	397823	7.3532%	782679	7.4473%	12967
Anaerococcus	1885	0.0370%	14186	0.2622%	16071	0.1529%	12301
Solobacterium	40703	0.7982%	52497	0.9703%	93200	0.8868%	11794
Ruminococcaceae_UCG-014	14116	0.2768%	25765	0.4762%	39881	0.3795%	11649
Campylobacter	65811	1.2906%	77151	1.4260%	142962	1.3603%	11340
Capnocytophaga	17866	0.3504%	26116	0.4827%	43982	0.4185%	8250
Parvimonas	12595	0.2470%	19664	0.3635%	32259	0.3069%	7069
Finegoldia	2213	0.0434%	5726	0.1058%	7939	0.0755%	3513
unidentified_Lachnospiraceae	6755	0.1325%	10253	0.1895%	17008	0.1618%	3498
Actinobacillus	57930	1.1360%	61004	1.1276%	118934	1.1317%	3074
Novosphingobium	553	0.0108%	3111	0.0575%	3664	0.0349%	2558
Shewanella	1315	0.0258%	3597	0.0665%	4912	0.0467%	2282
Thiovirga	1	0.0000%	2279	0.0421%	2280	0.0217%	2278
Eubacterium_nodatum_group	25003	0.4903%	27078	0.5005%	52081	0.4956%	2075

unidentified_Mitochondria	328	0.0064%	2032	0.0376%	2360	0.0225%	1704
unidentified_Candidate_division_SR1	9567	0.1876%	11138	0.2059%	20705	0.1970%	1571
Bacteroides	1612	0.0316%	3165	0.0585%	4777	0.0455%	1553
Propionibacterium	2823	0.0554%	3907	0.0722%	6730	0.0640%	1084
Thauera	12	0.0002%	1013	0.0187%	1025	0.0098%	1001
Pseudomonas	1604	0.0315%	2471	0.0457%	4075	0.0388%	867
Leptolyngbya	2	0.0000%	841	0.0155%	843	0.0080%	839
Kocuria	1248	0.0245%	2085	0.0385%	3333	0.0317%	837
Vibrio	223	0.0044%	973	0.0180%	1196	0.0114%	750
Blastocatella	66	0.0013%	806	0.0149%	872	0.0083%	740
Dialister	5183	0.1016%	5864	0.1084%	11047	0.1051%	681
Bacillus	375	0.0074%	1006	0.0186%	1381	0.0131%	631
Ensifer	86	0.0017%	689	0.0127%	775	0.0074%	603
Acinetobacter	2460	0.0482%	3060	0.0566%	5520	0.0525%	600
unidentified_WCHB1-69	2478	0.0486%	3066	0.0567%	5544	0.0528%	588
Arthrobacter	1868	0.0366%	2424	0.0448%	4292	0.0408%	556
unidentified_Veillonellaceae	1143	0.0224%	1679	0.0310%	2822	0.0269%	536

Faecalibacterium	213	0.0042%	726	0.0134%	939	0.0089%	513
Bdellovibrio	4	0.0001%	517	0.0096%	521	0.0050%	513
Lysobacter	31	0.0006%	527	0.0097%	558	0.0053%	496
Negativicoccus	454	0.0089%	917	0.0169%	1371	0.0130%	463
Steroidobacter	74	0.0015%	526	0.0097%	600	0.0057%	452
Chryseolinea	34	0.0007%	485	0.0090%	519	0.0049%	451
Limnobacter	4	0.0001%	437	0.0081%	441	0.0042%	433
Luteimonas	3	0.0001%	411	0.0076%	414	0.0039%	408
Haliangium	32	0.0006%	424	0.0078%	456	0.0043%	392
Pseudoxanthomonas	14	0.0003%	402	0.0074%	416	0.0040%	388
unidentified_Rhodospirillaceae	83	0.0016%	460	0.0085%	543	0.0052%	377
Paracocccus	367	0.0072%	737	0.0136%	1104	0.0105%	370
Sphingobium	1	0.0000%	371	0.0069%	372	0.0035%	370
Chlorobaculum	0	0.0000%	369	0.0068%	369	0.0035%	369
Sulfuricurvum	16	0.0003%	383	0.0071%	399	0.0038%	367
Methylotenera	47	0.0009%	408	0.0075%	455	0.0043%	361
Bergeyella	3009	0.0590%	3359	0.0621%	6368	0.0606%	350

Thiobacillus	28	0.0005%	378	0.0070%	406	0.0039%	350
Agromyces	48	0.0009%	390	0.0072%	438	0.0042%	342
Desulfuromonas	1	0.0000%	335	0.0062%	336	0.0032%	334
Parabacteroides	333	0.0065%	656	0.0121%	989	0.0094%	323
Methylophilus	91	0.0018%	408	0.0075%	499	0.0047%	317
Mogibacterium	2569	0.0504%	2880	0.0532%	5449	0.0518%	311
Aggregatibacter	28547	0.5598%	28855	0.5333%	57402	0.5462%	308
Arcobacter	74	0.0015%	374	0.0069%	448	0.0043%	300
Woodsholea	4	0.0001%	291	0.0054%	295	0.0028%	287
Streptomyces	121	0.0024%	399	0.0074%	520	0.0049%	278
Lactococcus	663	0.0130%	937	0.0173%	1600	0.0152%	274
Rhodanobacter	0	0.0000%	273	0.0050%	273	0.0026%	273
Mycobacterium	6	0.0001%	274	0.0051%	280	0.0027%	268
Raoultella	801	0.0157%	1066	0.0197%	1867	0.0178%	265
vadinBC27_wastewater-sludge_group	0	0.0000%	257	0.0048%	257	0.0024%	257
Mycoplasma	1019	0.0200%	1266	0.0234%	2285	0.0217%	247
Rhodobacter	5	0.0001%	248	0.0046%	253	0.0024%	243

Thioalkalimicrobium	0	0.0000%	238	0.0044%	238	0.0023%	238
Turicibacter	31	0.0006%	268	0.0050%	299	0.0028%	237
Devosia	69	0.0014%	302	0.0056%	371	0.0035%	233
Parvibaculum	1	0.0000%	229	0.0042%	230	0.0022%	228
Bryobacter	107	0.0021%	332	0.0061%	439	0.0042%	225
Candidatus_Arthromitus	61	0.0012%	284	0.0052%	345	0.0033%	223
Ezakiella	272	0.0053%	491	0.0091%	763	0.0073%	219
Trichococcus	58	0.0011%	277	0.0051%	335	0.0032%	219
Amaricoccus	50	0.0010%	267	0.0049%	317	0.0030%	217
Mobiluncus	311	0.0061%	523	0.0097%	834	0.0079%	212
Buchnera	37	0.0007%	241	0.0045%	278	0.0026%	204
Abiotrophia	1262	0.0247%	1464	0.0271%	2726	0.0259%	202
Family_XIII_UCG-001	1620	0.0318%	1818	0.0336%	3438	0.0327%	198
Cellulosimicrobium	136	0.0027%	334	0.0062%	470	0.0045%	198
Proteus	25	0.0005%	223	0.0041%	248	0.0024%	198
Terrimonas	2	0.0000%	195	0.0036%	197	0.0019%	193
Christensenellaceae_R-7_group	149	0.0029%	334	0.0062%	483	0.0046%	185

Ferruginibacter	5	0.0001%	190	0.0035%	195	0.0019%	185
Pseudobutyrivibrio	224	0.0044%	408	0.0075%	632	0.0060%	184
Rhizobium	294	0.0058%	477	0.0088%	771	0.0073%	183
Gaiella	59	0.0012%	240	0.0044%	299	0.0028%	181
Anaerotruncus	45	0.0009%	226	0.0042%	271	0.0026%	181
unidentified_Family_XIII	2208	0.0433%	2386	0.0441%	4594	0.0437%	178
Flavobacterium	65	0.0013%	239	0.0044%	304	0.0029%	174
Arenimonas	16	0.0003%	189	0.0035%	205	0.0020%	173
Hyphomicrobium	33	0.0006%	205	0.0038%	238	0.0023%	172
Rheinheimera	24	0.0005%	196	0.0036%	220	0.0021%	172
Romboutsia	190	0.0037%	361	0.0067%	551	0.0052%	171
Altererythrobacter	13	0.0003%	183	0.0034%	196	0.0019%	170
Sulfuritalea	2	0.0000%	171	0.0032%	173	0.0016%	169
Nocardioides	153	0.0030%	319	0.0059%	472	0.0045%	166
Macrococcus	12	0.0002%	173	0.0032%	185	0.0018%	161
unidentified_Nitrospiraceae	26	0.0005%	183	0.0034%	209	0.0020%	157
Halomonas	1325	0.0260%	1480	0.0274%	2805	0.0267%	155

Denitratisoma	6	0.0001%	160	0.0030%	166	0.0016%	154
Owenweeksia	0	0.0000%	149	0.0028%	149	0.0014%	149
unidentified_Gemmatimonadetes	96	0.0019%	243	0.0045%	339	0.0032%	147
Marmoricola	174	0.0034%	318	0.0059%	492	0.0047%	144
Sulfurovum	56	0.0011%	196	0.0036%	252	0.0024%	140
Nitrosomonas	4	0.0001%	143	0.0026%	147	0.0014%	139
Ruminococcaceae_UCG-002	28	0.0005%	166	0.0031%	194	0.0018%	138
Peredibacter	3	0.0001%	141	0.0026%	144	0.0014%	138
Sulfurimonas	0	0.0000%	136	0.0025%	136	0.0013%	136
unidentified_Ruminococcaceae	54	0.0011%	189	0.0035%	243	0.0023%	135
Aquicella	10	0.0002%	143	0.0026%	153	0.0015%	133
Solirubrobacter	41	0.0008%	172	0.0032%	213	0.0020%	131
Nordella	1	0.0000%	132	0.0024%	133	0.0013%	131
Oceanicella	3	0.0001%	131	0.0024%	134	0.0013%	128
Iamia	18	0.0004%	145	0.0027%	163	0.0016%	127
Fusicatenibacter	21	0.0004%	147	0.0027%	168	0.0016%	126
Butyrivibrio_2	3890	0.0763%	4013	0.0742%	7903	0.0752%	123

Variibacter	51	0.0010%	174	0.0032%	225	0.0021%	123
Paraprevotella	4	0.0001%	123	0.0023%	127	0.0012%	119
Massilia	411	0.0081%	528	0.0098%	939	0.0089%	117
Lachnospira	34	0.0007%	151	0.0028%	185	0.0018%	117
Adhaeribacter	36	0.0007%	151	0.0028%	187	0.0018%	115
Dechloromonas	21	0.0004%	132	0.0024%	153	0.0015%	111
Aminobacter	74	0.0015%	184	0.0034%	258	0.0025%	110
Alishewanella	0	0.0000%	110	0.0020%	110	0.0010%	110
Ornithinimicrobium	77	0.0015%	179	0.0033%	256	0.0024%	102
unidentified_GR-WP33-30	7	0.0001%	107	0.0020%	114	0.0011%	100
Defluviicoccus	49	0.0010%	147	0.0027%	196	0.0019%	98
Desulfomicrobium	26	0.0005%	124	0.0023%	150	0.0014%	98
Sphingosinicella	13	0.0003%	110	0.0020%	123	0.0012%	97
Anaerovorax	0	0.0000%	93	0.0017%	93	0.0009%	93
Agrococcus	367	0.0072%	459	0.0085%	826	0.0079%	92
Frondihabitans	318	0.0062%	410	0.0076%	728	0.0069%	92
Planomicrobium	471	0.0092%	562	0.0104%	1033	0.0098%	91

unidentified_Nitrosomonadaceae	10	0.0002%	99	0.0018%	109	0.0010%	89
Ohtaekwangia	1	0.0000%	90	0.0017%	91	0.0009%	89
Actinomadura	1	0.0000%	90	0.0017%	91	0.0009%	89
Illumatobacter	7	0.0001%	95	0.0018%	102	0.0010%	88
Dokdonella	18	0.0004%	105	0.0019%	123	0.0012%	87
Candidatus_Odyssella	3	0.0001%	90	0.0017%	93	0.0009%	87
Nannocystis	2	0.0000%	89	0.0016%	91	0.0009%	87
unidentified_Mollicutes_RF9	87	0.0017%	172	0.0032%	259	0.0025%	85
Nitrosospira	14	0.0003%	99	0.0018%	113	0.0011%	85
Candidatus_Alysiosphaera	11	0.0002%	96	0.0018%	107	0.0010%	85
Sphaerochaeta	14	0.0003%	98	0.0018%	112	0.0011%	84
hgcI_clade	5	0.0001%	89	0.0016%	94	0.0009%	84
Microbacterium	122	0.0024%	205	0.0038%	327	0.0031%	83
Blautia	46	0.0009%	129	0.0024%	175	0.0017%	83
Aeromicrobium	45	0.0009%	128	0.0024%	173	0.0016%	83
Bauldia	8	0.0002%	89	0.0016%	97	0.0009%	81
Ferritrophicum	2	0.0000%	82	0.0015%	84	0.0008%	80

Ruminococcaceae_UCG-005	81	0.0016%	159	0.0029%	240	0.0023%	78
Pelagibius	0	0.0000%	78	0.0014%	78	0.0007%	78
Skermanella	49	0.0010%	126	0.0023%	175	0.0017%	77
Burkholderia	199	0.0039%	274	0.0051%	473	0.0045%	75
Sorangium	3	0.0001%	78	0.0014%	81	0.0008%	75
Polynucleobacter	0	0.0000%	74	0.0014%	74	0.0007%	74
Rhizomicrobium	1	0.0000%	74	0.0014%	75	0.0007%	73
Reyranella	87	0.0017%	159	0.0029%	246	0.0023%	72
unidentified_Cytophagaceae	1	0.0000%	72	0.0013%	73	0.0007%	71
Sphingopyxis	13	0.0003%	82	0.0015%	95	0.0009%	69
Ruminococcaceae_UCG-003	7	0.0001%	76	0.0014%	83	0.0008%	69
Perlucidibaca	5	0.0001%	74	0.0014%	79	0.0008%	69
Serratia	244	0.0048%	312	0.0058%	556	0.0053%	68
Paenibacillus	32	0.0006%	100	0.0018%	132	0.0013%	68
Sulfurospirillum	11	0.0002%	79	0.0015%	90	0.0009%	68
Caulobacter	40	0.0008%	107	0.0020%	147	0.0014%	67
Lactobacillus	1578	0.0309%	1644	0.0304%	3222	0.0307%	66

Photobacterium	33	0.0006%	99	0.0018%	132	0.0013%	66
unidentified_Xanthomonadaceae	1	0.0000%	67	0.0012%	68	0.0006%	66
Leucobacter	57	0.0011%	121	0.0022%	178	0.0017%	64
Gardnerella	11	0.0002%	74	0.0014%	85	0.0008%	63
Pseudospirillum	0	0.0000%	63	0.0012%	63	0.0006%	63
Saccharopolyspora	14	0.0003%	76	0.0014%	90	0.0009%	62
Psychrobacillus	8	0.0002%	70	0.0013%	78	0.0007%	62
Candidatus_Microthrix	1	0.0000%	63	0.0012%	64	0.0006%	62
Hirschia	1	0.0000%	62	0.0011%	63	0.0006%	61
Chryseobacterium	88	0.0017%	147	0.0027%	235	0.0022%	59
Candidatus_Competibacter	6	0.0001%	65	0.0012%	71	0.0007%	59
unidentified_TRA3-20	0	0.0000%	59	0.0011%	59	0.0006%	59
Enterococcus	114	0.0022%	172	0.0032%	286	0.0027%	58
Ruegeria	4	0.0001%	62	0.0011%	66	0.0006%	58
unidentified_Syntrophaceae	0	0.0000%	57	0.0011%	57	0.0005%	57
Allobaculum	44	0.0009%	99	0.0018%	143	0.0014%	55
Facklamia	22	0.0004%	76	0.0014%	98	0.0009%	54

Salinicoccus	6	0.0001%	60	0.0011%	66	0.0006%	54
Helicobacter	97	0.0019%	150	0.0028%	247	0.0024%	53
Acidibacter	17	0.0003%	69	0.0013%	86	0.0008%	52
Flexivirga	12	0.0002%	64	0.0012%	76	0.0007%	52
Xanthomonas	8	0.0002%	60	0.0011%	68	0.0006%	52
Cobetia	2	0.0000%	54	0.0010%	56	0.0005%	52
Ruminococcaceae_UCG-010	135	0.0026%	184	0.0034%	319	0.0030%	49
Fluviicola	3	0.0001%	52	0.0010%	55	0.0005%	49
Filimonas	0	0.0000%	49	0.0009%	49	0.0005%	49
unidentified_Chitinophagaceae	1	0.0000%	49	0.0009%	50	0.0005%	48
Blastococcus	195	0.0038%	242	0.0045%	437	0.0042%	47
Azospirillum	24	0.0005%	71	0.0013%	95	0.0009%	47
unidentified_Thermomicrobia	7	0.0001%	53	0.0010%	60	0.0006%	46
Tamlana	0	0.0000%	46	0.0009%	46	0.0004%	46
Erysipelotrichaceae_UCG-003	0	0.0000%	46	0.0009%	46	0.0004%	46
Oscillibacter	5	0.0001%	50	0.0009%	55	0.0005%	45
Phaeodactylibacter	2	0.0000%	47	0.0009%	49	0.0005%	45

Wolinella	107	0.0021%	151	0.0028%	258	0.0025%	44
Odoribacter	95	0.0019%	139	0.0026%	234	0.0022%	44
Pseudonocardia	42	0.0008%	86	0.0016%	128	0.0012%	44
Erythrobacter	17	0.0003%	61	0.0011%	78	0.0007%	44
Pseudorhodoferax	1	0.0000%	45	0.0008%	46	0.0004%	44
Murdochiella	43	0.0008%	86	0.0016%	129	0.0012%	43
Ureaplasma	7	0.0001%	50	0.0009%	57	0.0005%	43
Brochothrix	28	0.0005%	70	0.0013%	98	0.0009%	42
Intestinimonas	26	0.0005%	68	0.0013%	94	0.0009%	42
Patulibacter	5	0.0001%	47	0.0009%	52	0.0005%	42
Methyloversatilis	2	0.0000%	44	0.0008%	46	0.0004%	42
Akkermansia	12	0.0002%	53	0.0010%	65	0.0006%	41
unidentified_OPB56	0	0.0000%	41	0.0008%	41	0.0004%	41
Subdoligranulum	66	0.0013%	106	0.0020%	172	0.0016%	40
Geodermatophilus	16	0.0003%	56	0.0010%	72	0.0007%	40
Phaselicystis	15	0.0003%	55	0.0010%	70	0.0007%	40
dgA-11_gut_group	4	0.0001%	44	0.0008%	48	0.0005%	40

Dietzia	86	0.0017%	125	0.0023%	211	0.0020%	39
Clostridium_sensu_stricto_1	283	0.0055%	321	0.0059%	604	0.0057%	38
Candidatus_Nitrotoga	16	0.0003%	54	0.0010%	70	0.0007%	38
Aliivibrio	1	0.0000%	39	0.0007%	40	0.0004%	38
Smithella	0	0.0000%	38	0.0007%	38	0.0004%	38
Pedobacter	91	0.0018%	128	0.0024%	219	0.0021%	37
unidentified_Acidobacteria	0	0.0000%	37	0.0007%	37	0.0004%	37
Stella	0	0.0000%	37	0.0007%	37	0.0004%	37
Eggerthella	1	0.0000%	37	0.0007%	38	0.0004%	36
Rubritalea	0	0.0000%	36	0.0007%	36	0.0003%	36
CL500-29_marine_group	4	0.0001%	38	0.0007%	42	0.0004%	34
Pedomicrobium	134	0.0026%	167	0.0031%	301	0.0029%	33
Flavonifractor	96	0.0019%	129	0.0024%	225	0.0021%	33
Cryptobacterium	33	0.0006%	66	0.0012%	99	0.0009%	33
Roseomonas	18	0.0004%	51	0.0009%	69	0.0007%	33
Albidiferax	12	0.0002%	45	0.0008%	57	0.0005%	33
Proteiniclasticum	7	0.0001%	40	0.0007%	47	0.0004%	33

Macellibacteroides	1	0.0000%	34	0.0006%	35	0.0003%	33
Truepera	0	0.0000%	33	0.0006%	33	0.0003%	33
Delftia	81	0.0016%	113	0.0021%	194	0.0018%	32
Candidatus_Entotheonella	8	0.0002%	40	0.0007%	48	0.0005%	32
Marinicella	0	0.0000%	32	0.0006%	32	0.0003%	32
Candidatus_Solibacter	46	0.0009%	77	0.0014%	123	0.0012%	31
Piscinibacter	4	0.0001%	35	0.0006%	39	0.0004%	31
Azoarcus	2	0.0000%	33	0.0006%	35	0.0003%	31
Ferrovibrio	1	0.0000%	32	0.0006%	33	0.0003%	31
Syntrophomonas	0	0.0000%	31	0.0006%	31	0.0003%	31
Bosea	75	0.0015%	105	0.0019%	180	0.0017%	30
Methylomonas	1	0.0000%	31	0.0006%	32	0.0003%	30
Solimonas	0	0.0000%	30	0.0006%	30	0.0003%	30
Desulfococcus	0	0.0000%	30	0.0006%	30	0.0003%	30
Craurococcus	0	0.0000%	30	0.0006%	30	0.0003%	30
Deinococcus	55	0.0011%	84	0.0016%	139	0.0013%	29
Lachnospiraceae_UCG-010	10	0.0002%	39	0.0007%	49	0.0005%	29

Rhodopila	3	0.0001%	32	0.0006%	35	0.0003%	29
Luedemannella	2	0.0000%	31	0.0006%	33	0.0003%	29
Marinilactibacillus	1	0.0000%	30	0.0006%	31	0.0003%	29
Pannonibacter	95	0.0019%	123	0.0023%	218	0.0021%	28
Modestobacter	22	0.0004%	50	0.0009%	72	0.0007%	28
Tessaracoccus	8	0.0002%	36	0.0007%	44	0.0004%	28
Amycolatopsis	7	0.0001%	35	0.0006%	42	0.0004%	28
Parapusillimonas	0	0.0000%	28	0.0005%	28	0.0003%	28
Desulforegula	0	0.0000%	28	0.0005%	28	0.0003%	28
Candidatus_Captivus	0	0.0000%	28	0.0005%	28	0.0003%	28
Stenotrophomonas	255	0.0050%	281	0.0052%	536	0.0051%	26
Clostridium_sensu_stricto_10	16	0.0003%	42	0.0008%	58	0.0006%	26
Erysipelothrix	9	0.0002%	35	0.0006%	44	0.0004%	26
Salana	6	0.0001%	30	0.0006%	36	0.0003%	24
Pontibacter	29	0.0006%	52	0.0010%	81	0.0008%	23
Asteroleplasma	15	0.0003%	38	0.0007%	53	0.0005%	23
Sporacetigenium	2	0.0000%	25	0.0005%	27	0.0003%	23

Sinobaca	1	0.0000%	24	0.0004%	25	0.0002%	23
Chthoniobacter	1	0.0000%	24	0.0004%	25	0.0002%	23
Blvii28_wastewater-sludge_group	0	0.0000%	23	0.0004%	23	0.0002%	23
Nonomuraea	9	0.0002%	31	0.0006%	40	0.0004%	22
Kaistia	7	0.0001%	29	0.0005%	36	0.0003%	22
unidentified_Gaiellales	6	0.0001%	28	0.0005%	34	0.0003%	22
Gemmobacter	6	0.0001%	28	0.0005%	34	0.0003%	22
Haloactinopolyspora	1	0.0000%	23	0.0004%	24	0.0002%	22
unidentified_Woesearchaeota_.DHVEG-6.	0	0.0000%	22	0.0004%	22	0.0002%	22
unidentified_Actinobacteria	0	0.0000%	22	0.0004%	22	0.0002%	22
Lentibacillus	0	0.0000%	22	0.0004%	22	0.0002%	22
Hungatella	32	0.0006%	53	0.0010%	85	0.0008%	21
unidentified_Methylophilaceae	8	0.0002%	29	0.0005%	37	0.0004%	21
Parvularcula	2	0.0000%	23	0.0004%	25	0.0002%	21
Mesoplasma	2	0.0000%	23	0.0004%	25	0.0002%	21
Marixanthomonas	1	0.0000%	22	0.0004%	23	0.0002%	21

Denitromonas	1	0.0000%	22	0.0004%	23	0.0002%	21
Thermomonas	0	0.0000%	21	0.0004%	21	0.0002%	21
Polycyclovorans	0	0.0000%	21	0.0004%	21	0.0002%	21
Aminivibrio	0	0.0000%	21	0.0004%	21	0.0002%	21
Pseudoramibacter	122	0.0024%	142	0.0026%	264	0.0025%	20
Promicromonospora	24	0.0005%	44	0.0008%	68	0.0006%	20
Aerococcus	22	0.0004%	42	0.0008%	64	0.0006%	20
Parascardovia	13	0.0003%	33	0.0006%	46	0.0004%	20
Acidovorax	13	0.0003%	33	0.0006%	46	0.0004%	20
Ruminococcaceae_UCG-009	9	0.0002%	29	0.0005%	38	0.0004%	20
Kribbella	3	0.0001%	23	0.0004%	26	0.0002%	20
Rhizocola	0	0.0000%	20	0.0004%	20	0.0002%	20
Family_XIII_UCG-002	0	0.0000%	20	0.0004%	20	0.0002%	20
Constrictibacter	0	0.0000%	20	0.0004%	20	0.0002%	20
Rs-M59_termite_group	35	0.0007%	54	0.0010%	89	0.0008%	19
Pseudoduganella	19	0.0004%	38	0.0007%	57	0.0005%	19
Oceanobacillus	9	0.0002%	28	0.0005%	37	0.0004%	19

Pelagibacterium	1	0.0000%	20	0.0004%	21	0.0002%	19
Coprococcus	100	0.0020%	118	0.0022%	218	0.0021%	18
Rubrobacter	82	0.0016%	100	0.0018%	182	0.0017%	18
Lawsonia	12	0.0002%	30	0.0006%	42	0.0004%	18
Methyloceanibacter	1	0.0000%	19	0.0004%	20	0.0002%	18
Granulicella	1	0.0000%	19	0.0004%	20	0.0002%	18
Agaricicola	1	0.0000%	19	0.0004%	20	0.0002%	18
Roseobacter_clade_CHAB-I-5_lineage	0	0.0000%	18	0.0003%	18	0.0002%	18
Pseudolabrys	0	0.0000%	18	0.0003%	18	0.0002%	18
Gallicola	0	0.0000%	18	0.0003%	18	0.0002%	18
Jeotgalicoccus	65	0.0013%	82	0.0015%	147	0.0014%	17
Nesterenkonia	25	0.0005%	42	0.0008%	67	0.0006%	17
Prevotellaceae_Ga6A1_group	4	0.0001%	21	0.0004%	25	0.0002%	17
Dongia	4	0.0001%	21	0.0004%	25	0.0002%	17
Elstera	0	0.0000%	17	0.0003%	17	0.0002%	17
Erysipelotrichaceae_UCG-006	157	0.0031%	173	0.0032%	330	0.0031%	16
Cetobacterium	25	0.0005%	41	0.0008%	66	0.0006%	16

Taibaiella	13	0.0003%	29	0.0005%	42	0.0004%	16
Thiorhodospira	3	0.0001%	19	0.0004%	22	0.0002%	16
Cytophaga	3	0.0001%	19	0.0004%	22	0.0002%	16
Sandaracinus	2	0.0000%	18	0.0003%	20	0.0002%	16
Prosthecobacter	1	0.0000%	17	0.0003%	18	0.0002%	16
Planifilum	0	0.0000%	16	0.0003%	16	0.0002%	16
Aquimonas	0	0.0000%	16	0.0003%	16	0.0002%	16
Gordonia	6	0.0001%	21	0.0004%	27	0.0003%	15
Sporomusa	3	0.0001%	18	0.0003%	21	0.0002%	15
Acidiferrobacter	0	0.0000%	15	0.0003%	15	0.0001%	15
Acetobacterium	3	0.0001%	17	0.0003%	20	0.0002%	14
Desulfocapsa	1	0.0000%	15	0.0003%	16	0.0002%	14
Cohnella	1	0.0000%	15	0.0003%	16	0.0002%	14
Meiothermus	0	0.0000%	14	0.0003%	14	0.0001%	14
Gracilibacillus	0	0.0000%	14	0.0003%	14	0.0001%	14
Atopostipes	0	0.0000%	14	0.0003%	14	0.0001%	14
unidentified_Anaerolineaceae	5	0.0001%	18	0.0003%	23	0.0002%	13

Xanthobacter	1	0.0000%	14	0.0003%	15	0.0001%	13
Varibaculum	1	0.0000%	14	0.0003%	15	0.0001%	13
Bacteriovorax	1	0.0000%	14	0.0003%	15	0.0001%	13
SM1A02	0	0.0000%	13	0.0002%	13	0.0001%	13
Methylocaldum	0	0.0000%	13	0.0002%	13	0.0001%	13
Marinomonas	7	0.0001%	19	0.0004%	26	0.0002%	12
Propioniciclava	4	0.0001%	16	0.0003%	20	0.0002%	12
Eubacterium_ventriosum_group	4	0.0001%	16	0.0003%	20	0.0002%	12
Diaphorobacter	4	0.0001%	16	0.0003%	20	0.0002%	12
Lachnospiraceae_UCG-004	2	0.0000%	14	0.0003%	16	0.0002%	12
Acidothermus	2	0.0000%	14	0.0003%	16	0.0002%	12
Undibacterium	1	0.0000%	13	0.0002%	14	0.0001%	12
Labrys	1	0.0000%	13	0.0002%	14	0.0001%	12
Alkalibacterium	1	0.0000%	13	0.0002%	14	0.0001%	12
Treponema	0	0.0000%	12	0.0002%	12	0.0001%	12
Desulfomonile	0	0.0000%	12	0.0002%	12	0.0001%	12
Sporosarcina	30	0.0006%	41	0.0008%	71	0.0007%	11

Anoxybacillus	22	0.0004%	33	0.0006%	55	0.0005%	11
Duganella	5	0.0001%	16	0.0003%	21	0.0002%	11
Sedimenticola	4	0.0001%	15	0.0003%	19	0.0002%	11
Friedmanniella	4	0.0001%	15	0.0003%	19	0.0002%	11
Cryptanaerobacter	1	0.0000%	12	0.0002%	13	0.0001%	11
unidentified_Rickettsiaceae	0	0.0000%	11	0.0002%	11	0.0001%	11
unidentified_Halothiobacillaceae	0	0.0000%	11	0.0002%	11	0.0001%	11
Saccharomonospora	0	0.0000%	11	0.0002%	11	0.0001%	11
Hydrogenoanaerobacterium	0	0.0000%	11	0.0002%	11	0.0001%	11
Desulfobacter	0	0.0000%	11	0.0002%	11	0.0001%	11
Paucisalibacillus	19	0.0004%	29	0.0005%	48	0.0005%	10
unidentified_Acidimicrobiales	9	0.0002%	19	0.0004%	28	0.0003%	10
Parafilimonas	8	0.0002%	18	0.0003%	26	0.0002%	10
Solibacillus	7	0.0001%	17	0.0003%	24	0.0002%	10
Senegalimassilia	4	0.0001%	14	0.0003%	18	0.0002%	10
Desulfatiglans	4	0.0001%	14	0.0003%	18	0.0002%	10
Actinomycetospora	1	0.0000%	11	0.0002%	12	0.0001%	10

Syntrophus	0	0.0000%	10	0.0002%	10	0.0001%	10
Pseudanabaena	0	0.0000%	10	0.0002%	10	0.0001%	10
Filomicrobium	0	0.0000%	10	0.0002%	10	0.0001%	10
Fictibacillus	0	0.0000%	10	0.0002%	10	0.0001%	10
Empedobacter	9	0.0002%	18	0.0003%	27	0.0003%	9
Simiduia	8	0.0002%	17	0.0003%	25	0.0002%	9
Clostridium_sensu_stricto_3	7	0.0001%	16	0.0003%	23	0.0002%	9
Desulfobacca	5	0.0001%	14	0.0003%	19	0.0002%	9
Myroides	2	0.0000%	11	0.0002%	13	0.0001%	9
Haloferula	2	0.0000%	11	0.0002%	13	0.0001%	9
Microcoleus	1	0.0000%	10	0.0002%	11	0.0001%	9
Vitellibacter	0	0.0000%	9	0.0002%	9	0.0001%	9
Nosocomiicoccus	0	0.0000%	9	0.0002%	9	0.0001%	9
Inquilinus	0	0.0000%	9	0.0002%	9	0.0001%	9
Flectobacillus	0	0.0000%	9	0.0002%	9	0.0001%	9
Anaerobiospirillum	0	0.0000%	9	0.0002%	9	0.0001%	9
Cellvibrio	71	0.0014%	79	0.0015%	150	0.0014%	8

Vagococcus	13	0.0003%	21	0.0004%	34	0.0003%	8
Gillisia	7	0.0001%	15	0.0003%	22	0.0002%	8
Epulopiscium	1	0.0000%	9	0.0002%	10	0.0001%	8
Coprobacter	1	0.0000%	9	0.0002%	10	0.0001%	8
unidentified_34P16	0	0.0000%	8	0.0001%	8	0.0001%	8
Sphaerobacter	0	0.0000%	8	0.0001%	8	0.0001%	8
Myxococcus	0	0.0000%	8	0.0001%	8	0.0001%	8
Melghirimyces	0	0.0000%	8	0.0001%	8	0.0001%	8
Candidatus_Caldatribacterium	0	0.0000%	8	0.0001%	8	0.0001%	8
Brachyspira	0	0.0000%	8	0.0001%	8	0.0001%	8
Acetoanaerobium	0	0.0000%	8	0.0001%	8	0.0001%	8
Sva0081_sediment_group	1	0.0000%	8	0.0001%	9	0.0001%	7
Nocardia	1	0.0000%	8	0.0001%	9	0.0001%	7
Kineococcus	1	0.0000%	8	0.0001%	9	0.0001%	7
Corallococcus	1	0.0000%	8	0.0001%	9	0.0001%	7
Solitalea	0	0.0000%	7	0.0001%	7	0.0001%	7
Rhodocytophaga	0	0.0000%	7	0.0001%	7	0.0001%	7

Fontimonas	0	0.0000%	7	0.0001%	7	0.0001%	7
Arcanobacterium	0	0.0000%	7	0.0001%	7	0.0001%	7
Algoriphagus	60	0.0012%	66	0.0012%	126	0.0012%	6
Gemmatimonas	46	0.0009%	52	0.0010%	98	0.0009%	6
Acetitomaculum	14	0.0003%	20	0.0004%	34	0.0003%	6
Dermabacter	8	0.0002%	14	0.0003%	22	0.0002%	6
Opitutus	2	0.0000%	8	0.0001%	10	0.0001%	6
Family_XIII_AD3011_group	2	0.0000%	8	0.0001%	10	0.0001%	6
Bartonella	2	0.0000%	8	0.0001%	10	0.0001%	6
Vulgatibacter	1	0.0000%	7	0.0001%	8	0.0001%	6
Flavitalea	1	0.0000%	7	0.0001%	8	0.0001%	6
Tahibacter	0	0.0000%	6	0.0001%	6	0.0001%	6
Salinimicrobium	0	0.0000%	6	0.0001%	6	0.0001%	6
Methanolobus	0	0.0000%	6	0.0001%	6	0.0001%	6
Kroppenstedtia	0	0.0000%	6	0.0001%	6	0.0001%	6
Helcobacillus	0	0.0000%	6	0.0001%	6	0.0001%	6
Halothiobacillus	0	0.0000%	6	0.0001%	6	0.0001%	6

AUTHM297	0	0.0000%	6	0.0001%	6	0.0001%	6
Anaeromyxobacter	0	0.0000%	6	0.0001%	6	0.0001%	6
unidentified_Gracilibacteria	395	0.0077%	400	0.0074%	795	0.0076%	5
Phocaeicola	106	0.0021%	111	0.0021%	217	0.0021%	5
Collinsella	22	0.0004%	27	0.0005%	49	0.0005%	5
unidentified_Coriobacteriaceae	20	0.0004%	25	0.0005%	45	0.0004%	5
Silanimonas	17	0.0003%	22	0.0004%	39	0.0004%	5
Hydrogenophaga	4	0.0001%	9	0.0002%	13	0.0001%	5
unidentified_Hyphomicrobiaceae	3	0.0001%	8	0.0001%	11	0.0001%	5
Ignavibacterium	3	0.0001%	8	0.0001%	11	0.0001%	5
Luteococcus	2	0.0000%	7	0.0001%	9	0.0001%	5
Arcticibacter	2	0.0000%	7	0.0001%	9	0.0001%	5
Sedimentibacter	1	0.0000%	6	0.0001%	7	0.0001%	5
Nocardiopsis	1	0.0000%	6	0.0001%	7	0.0001%	5
Glaciecola	1	0.0000%	6	0.0001%	7	0.0001%	5
Zavarzinia	0	0.0000%	5	0.0001%	5	0.0000%	5
unidentified_OPB35_soil_group	0	0.0000%	5	0.0001%	5	0.0000%	5

unidentified_NKB5	0	0.0000%	5	0.0001%	5	0.0000%	5
unidentified_Moraxellaceae	0	0.0000%	5	0.0001%	5	0.0000%	5
Rhodomicrobium	0	0.0000%	5	0.0001%	5	0.0000%	5
Ornithobacterium	0	0.0000%	5	0.0001%	5	0.0000%	5
Novispirillum	0	0.0000%	5	0.0001%	5	0.0000%	5
Longispora	0	0.0000%	5	0.0001%	5	0.0000%	5
Gottschalkia	0	0.0000%	5	0.0001%	5	0.0000%	5
Elioraea	0	0.0000%	5	0.0001%	5	0.0000%	5
Conexibacter	0	0.0000%	5	0.0001%	5	0.0000%	5
Exiguobacterium	86	0.0017%	90	0.0017%	176	0.0017%	4
Thalassospira	20	0.0004%	24	0.0004%	44	0.0004%	4
Ruminococcaceae_UCG-013	12	0.0002%	16	0.0003%	28	0.0003%	4
Syntrophorhabdus	5	0.0001%	9	0.0002%	14	0.0001%	4
Marinococcus	5	0.0001%	9	0.0002%	14	0.0001%	4
Glycomyces	4	0.0001%	8	0.0001%	12	0.0001%	4
Candidatus_Chloroploca	3	0.0001%	7	0.0001%	10	0.0001%	4
Synergistes	2	0.0000%	6	0.0001%	8	0.0001%	4

Parapedobacter	2	0.0000%	6	0.0001%	8	0.0001%	4
Sulfuricella	1	0.0000%	5	0.0001%	6	0.0001%	4
Nakamurella	1	0.0000%	5	0.0001%	6	0.0001%	4
Clostridium_sensu_stricto_5	1	0.0000%	5	0.0001%	6	0.0001%	4
Vulcanibacillus	0	0.0000%	4	0.0001%	4	0.0000%	4
Subsaxibacter	0	0.0000%	4	0.0001%	4	0.0000%	4
Sporanaerobacter	0	0.0000%	4	0.0001%	4	0.0000%	4
Propionimicrobium	0	0.0000%	4	0.0001%	4	0.0000%	4
Microbispora	0	0.0000%	4	0.0001%	4	0.0000%	4
Desulfotomaculum	0	0.0000%	4	0.0001%	4	0.0000%	4
Dactylosporangium	0	0.0000%	4	0.0001%	4	0.0000%	4
Candidatus_Koribacter	0	0.0000%	4	0.0001%	4	0.0000%	4
Caldithrix	0	0.0000%	4	0.0001%	4	0.0000%	4
Arsenophonus	0	0.0000%	4	0.0001%	4	0.0000%	4
Anaerosalibacter	0	0.0000%	4	0.0001%	4	0.0000%	4
Arsenicicoccus	31	0.0006%	34	0.0006%	65	0.0006%	3
Pelospora	25	0.0005%	28	0.0005%	53	0.0005%	3

Propionivibrio	9	0.0002%	12	0.0002%	21	0.0002%	3
Pusillimonas	6	0.0001%	9	0.0002%	15	0.0001%	3
unidentified_0319-6A21	5	0.0001%	8	0.0001%	13	0.0001%	3
Anaerostipes	4	0.0001%	7	0.0001%	11	0.0001%	3
MSBL7	3	0.0001%	6	0.0001%	9	0.0001%	3
Methylobacter	3	0.0001%	6	0.0001%	9	0.0001%	3
Thermicanus	2	0.0000%	5	0.0001%	7	0.0001%	3
Ruminococcaceae_UCG-012	1	0.0000%	4	0.0001%	5	0.0000%	3
Papillibacter	1	0.0000%	4	0.0001%	5	0.0000%	3
Nitrolancea	1	0.0000%	4	0.0001%	5	0.0000%	3
Lutibacter	1	0.0000%	4	0.0001%	5	0.0000%	3
Lachnobacterium	1	0.0000%	4	0.0001%	5	0.0000%	3
Halioglobus	1	0.0000%	4	0.0001%	5	0.0000%	3
Eubacterium_hallii_group	1	0.0000%	4	0.0001%	5	0.0000%	3
Alkanindiges	1	0.0000%	4	0.0001%	5	0.0000%	3
unidentified_SJA-28	0	0.0000%	3	0.0001%	3	0.0000%	3
unidentified_Oligoflexaceae	0	0.0000%	3	0.0001%	3	0.0000%	3

unidentified_480-2	0	0.0000%	3	0.0001%	3	0.0000%	3
unidentified_4-15	0	0.0000%	3	0.0001%	3	0.0000%	3
Thermovirga	0	0.0000%	3	0.0001%	3	0.0000%	3
Synechococcus	0	0.0000%	3	0.0001%	3	0.0000%	3
Olivibacter	0	0.0000%	3	0.0001%	3	0.0000%	3
Nitrosococcus	0	0.0000%	3	0.0001%	3	0.0000%	3
Methylohalomonas	0	0.0000%	3	0.0001%	3	0.0000%	3
Holdemanella	0	0.0000%	3	0.0001%	3	0.0000%	3
Enterorhabdus	0	0.0000%	3	0.0001%	3	0.0000%	3
Cylindrospermosis	0	0.0000%	3	0.0001%	3	0.0000%	3
Brevinema	0	0.0000%	3	0.0001%	3	0.0000%	3
Ammoniibacillus	0	0.0000%	3	0.0001%	3	0.0000%	3
Alkaliphilus	0	0.0000%	3	0.0001%	3	0.0000%	3
Alcaligenes	0	0.0000%	3	0.0001%	3	0.0000%	3
Howardella	57	0.0011%	59	0.0011%	116	0.0011%	2
Coxiella	32	0.0006%	34	0.0006%	66	0.0006%	2
Legionella	15	0.0003%	17	0.0003%	32	0.0003%	2

Candidatus_Tammella	4	0.0001%	6	0.0001%	10	0.0001%	2
Tissierella	3	0.0001%	5	0.0001%	8	0.0001%	2
Luteolibacter	3	0.0001%	5	0.0001%	8	0.0001%	2
unidentified_SC-I-84	1	0.0000%	3	0.0001%	4	0.0000%	2
Proteiniphilum	1	0.0000%	3	0.0001%	4	0.0000%	2
Herpetosiphon	1	0.0000%	3	0.0001%	4	0.0000%	2
unidentified_Sphingobacteriaceae	0	0.0000%	2	0.0000%	2	0.0000%	2
unidentified_Alcaligenaceae	0	0.0000%	2	0.0000%	2	0.0000%	2
Turneriella	0	0.0000%	2	0.0000%	2	0.0000%	2
Salinivibrio	0	0.0000%	2	0.0000%	2	0.0000%	2
Psychrilyobacter	0	0.0000%	2	0.0000%	2	0.0000%	2
Nitriliruptor	0	0.0000%	2	0.0000%	2	0.0000%	2
Neochlamydia	0	0.0000%	2	0.0000%	2	0.0000%	2
Methanoregula	0	0.0000%	2	0.0000%	2	0.0000%	2
Iodobacter	0	0.0000%	2	0.0000%	2	0.0000%	2
Holdemania	0	0.0000%	2	0.0000%	2	0.0000%	2
Geothermobacter	0	0.0000%	2	0.0000%	2	0.0000%	2

Ferrimicrobium	0	0.0000%	2	0.0000%	2	0.0000%	2
Desulfovirga	0	0.0000%	2	0.0000%	2	0.0000%	2
Desulfitobacterium	0	0.0000%	2	0.0000%	2	0.0000%	2
Desulfitibacter	0	0.0000%	2	0.0000%	2	0.0000%	2
Denitrovibrio	0	0.0000%	2	0.0000%	2	0.0000%	2
Crocinitomix	0	0.0000%	2	0.0000%	2	0.0000%	2
Brachybacterium	213	0.0042%	214	0.0040%	427	0.0041%	1
Bifidobacterium	128	0.0025%	129	0.0024%	257	0.0024%	1
Georgenia	30	0.0006%	31	0.0006%	61	0.0006%	1
Hydrogenophilus	11	0.0002%	12	0.0002%	23	0.0002%	1
Mizugakiibacter	5	0.0001%	6	0.0001%	11	0.0001%	1
unidentified_Xanthomonadales	4	0.0001%	5	0.0001%	9	0.0001%	1
Syntrophococcus	4	0.0001%	5	0.0001%	9	0.0001%	1
Paludibacter	4	0.0001%	5	0.0001%	9	0.0001%	1
Mucispirillum	3	0.0001%	4	0.0001%	7	0.0001%	1
Acidaminococcus	3	0.0001%	4	0.0001%	7	0.0001%	1
Dorea	2	0.0000%	3	0.0001%	5	0.0000%	1

Acidaminobacter	2	0.0000%	3	0.0001%	5	0.0000%	1
Saccharibacillus	1	0.0000%	2	0.0000%	3	0.0000%	1
Nibribacter	1	0.0000%	2	0.0000%	3	0.0000%	1
Gelidibacter	1	0.0000%	2	0.0000%	3	0.0000%	1

Table S5. The original habitats of 142 new genera for post-smog swab included

Taxonomy	Original habitats	Reads in post-smog swabs	Being detceted in Tsinghua PM study	Number of carrying vendors
Planifilum	Fecal	16	yes	7
Atopostipes	Fecal	14	no	3
Treponema	Fecal	12	yes	2
Anaerobiospirillum	Fecal	9	yes	2
Brachyspira	Fecal	8	yes	1
Arcanobacterium	Fecal	7	yes	2
Gottschalkia	Fecal	5	no	2
Arsenophonus	Fecal	4	no	1
Enterorhabdus	Fecal	3	no	2
Holdemanella	Fecal	3	no	2
Ammoniibacillus	Fecal	3	no	3
Holdemania	Fecal	2	no	2
Polynucleobacter	Freshwater	74	yes	5
Filimonas	Freshwater	49	no	13
Desulforegula	Freshwater	28	no	11
Gracilibacillus	Freshwater	14	no	8
Pseudanabaena	Freshwater	10	yes	2
Rhodomicrobium	Freshwater	5	no	4
Zavarzinia	Freshwater	5	no	3
Desulfitibacter	Freshwater	2	no	1
Truepera	Hotspring	33	no	11
Aquimonas	Hotspring	16	no	7
Fictibacillus	Hotspring	10	no	5
Fontimonas	Hotspring	7	no	6
Elioraea	Hotspring	5	no	5
Synechococcus	Hotspring	3	yes	1
Geothermobacter	Hotspring	2	no	2
Owenweeksia	Marine	149	no	13
Pelagibius	Marine	78	no	14
Pseudospirillum	Marine	63	no	16
Tamlana	Marine	46	no	13
Rubritalea	Marine	36	no	2
Marinicella	Marine	32	no	13

Roseobacter_clade_CHAB-I-5_lineage	Marine	18	no	1
Methylocaldum	Marine	13	yes	4
Desulfomonile	Marine	12	no	7
Vitellibacter	Marine	9	no	6
Melghirimyces	Marine	8	no	7
Halothiobacillus	Marine	6	no	4
Subsaxibacter	Marine	4	no	3
Vulcanibacillus	Marine	4	no	4
Nitrosococcus	Marine	3	yes	3
Thermovirga	Marine	3	no	3
Iodobacter	Marine	2	no	1
Psychrilyobacter	Marine	2	no	2
Thioalkalimicrobium	Saline lake	238	no	15
Filomicrobium	Saline lake	10	no	5
Methylohalomonas	Saline lake	3	no	3
Nitriliruptor	Saline lake	2	no	2
Salinivibrio	Saline lake	2	no	2
Chlorobaculum	Sewage sludge	369	no	23
vadinBC27_wastewater-sludge_group	Sewage sludge	257	no	15
Syntrophomonas	Sewage sludge	31	yes	8
Parapusillimonas	Sewage sludge	28	no	11
Blvii28_wastewater-sludge_group	Sewage sludge	23	no	9
Hydrogenoanaerobacterium	Sewage sludge	11	no	5
Sphaerobacter	Sewage sludge	8	no	5
Tahibacter	Sewage sludge	6	no	4
Anaerosalibacter	Sewage sludge	4	no	4
Sporanaerobacter	Sewage sludge	4	yes	2
Brevinema	Sewage sludge	3	no	1
Desulfovirga	Sewage sludge	2	no	1
Rhodanobacter	Soil	273	yes	13
Sulfurimonas	Soil	136	no	15
Alishewanella	Soil	110	no	12
Anaerovorax	Soil	93	no	13
Smithella	Soil	38	no	13
Stella	Soil	37	no	14
Craurococcus	Soil	30	no	10
Desulfococcus	Soil	30	no	12
Solimonas	Soil	30	yes	6

Lentibacillus	Soil	22	yes	5
Aminivibrio	Soil	21	no	9
Thermomonas	Soil	21	yes	3
Constrictibacter	Soil	20	no	9
Rhizocola	Soil	20	no	9
Gallicola	Soil	18	no	8
Pseudolabrys	Soil	18	no	5
Elstera	Soil	17	no	1
Acidiferrobacter	Soil	15	no	8
Meiothermus	Soil	14	yes	3
Desulfobacter	Soil	11	yes	7
Saccharomonospora	Soil	11	yes	7
Inquilinus	Soil	9	yes	5
Flectobacillus	Soil	9	yes	1
Acetoanaerobium	Soil	8	no	8
Myxococcus	Soil	8	yes	5
Solitalea	Soil	7	no	5
Anaeromyxobacter	Soil	6	yes	4
Kroppenstedtia	Soil	6	no	2
Methanolobus	Soil	6	no	1
Salinimicrobium	Soil	6	yes	5
Conexibacter	Soil	5	no	3
Longispora	Soil	5	no	5
Novispirillum	Soil	5	no	3
Caldithrix	Soil	4	no	4
Dactylosporangium	Soil	4	yes	4
Desulfotomaculum	Soil	4	yes	3
Microbispora	Soil	4	yes	2
Alcaligenes	Soil	3	yes	1
Olivibacter	Soil	3	yes	1
Alkaliphilus	Soil	3	yes	3
Crocinitomix	Soil	2	no	2
Denitrovibrio	Soil	2	no	2
Desulfitobacterium	Soil	2	yes	1
Ferrimicrobium	Soil	2	yes	1
Methanoregula	Soil	2	yes	1
unidentified_TRA3-20	Uncertainty	59	no	16
unidentified_Syntrophaceae	Uncertainty	57	no	12

Erysipelotrichaceae_UCG-003	Uncertainty	46	no	5
unidentified_OPB56	Uncertainty	41	no	14
unidentified_Acidobacteria	Uncertainty	37	no	12
Candidatus_Captivus	Uncertainty	28	no	9
unidentified_Actinobacteria	Uncertainty	22	no	9
unidentified_Woesearchaeota_.DHVEG-6.	Uncertainty	22	no	11
Polycyclovorans	Uncertainty	21	no	11
Family_XIII_UCG-002	Uncertainty	20	no	8
SM1A02	Uncertainty	13	no	4
unidentified_Halothiobacillaceae	Uncertainty	11	no	7
unidentified_Rickettsiaceae	Uncertainty	11	no	7
Syntrophus	Uncertainty	10	yes	7
Nosocomiicoccus	Uncertainty	9	no	3
Candidatus_Caldatribacterium	Uncertainty	8	no	1
unidentified_34P16	Uncertainty	8	no	5
Rhodocytophaga	Uncertainty	7	no	3
Helcobacillus	Uncertainty	6	no	3
AUTHM297	Uncertainty	6	no	3
Ornithobacterium	Uncertainty	5	no	3
unidentified_Moraxellaceae	Uncertainty	5	no	3
unidentified_NKB5	Uncertainty	5	no	4
unidentified_OPB35_soil_group	Uncertainty	5	no	4
Propionimicrobium	Uncertainty	4	no	1
Candidatus_Koribacter	Uncertainty	4	yes	1
Cylindrospermosis	Uncertainty	3	no	1
unidentified_4-15	Uncertainty	3	no	2
unidentified_480-2	Uncertainty	3	no	3
unidentified_Oligoflexaceae	Uncertainty	3	no	3
unidentified_SJA-28	Uncertainty	3	no	3
Turneriella	Uncertainty	2	no	2
Neochlamydia	Uncertainty	2	no	2
unidentified_Alcaligenaceae	Uncertainty	2	no	2
unidentified_Sphingobacteriaceae	Uncertainty	2	no	2

Table S6. Alpha diversity of microbiota in pre- and post-smog nasopharynx swabs

Alpha diversity indices	pre-smog swabs	post-smog swabs	*P* value (Paired *t* test)
Number of observed species	379.1±120.0	484.3±239.5	0.000
Shannon	5.1781±0.6204	5.2890±0.6436	0.216
Simpson	0.9250±0.4346	0.9310±0.4910	0.409
Chao1	479.6±179.3	645.0±376.6	0.000
ACE	482.8±153.8	667.5±392.4	0.000
Whole-tree phylogenetic diversity	142.0±100.7	164.5±102.0	0.038

Table S7. The correlation between influencing factors and bacterial community structure of nasopharyngeal microbiota of 83 vendors

Influencing factors	RDA1	RDA2	r2	Pr (>r)
smog	0.984482	0.175487	0.186508	0.0005
gender	-0.37568	0.926747	0.120049	0.0005
working hour	-0.73229	0.680993	0.014857	0.287856
with masks	0.094388	-0.99554	0.056474	0.003498
smoking	-0.38828	0.921541	0.20441	0.0005
history of using antibiotics	-0.90232	0.431061	0.014139	0.303348
history of respiratory symptoms	0.751159	-0.66012	0.028437	0.097451

Table S8. Comparison of alpha diversity indexes of pre- and post-smog pharyngeal microbiota of people wearing masks

Alpha diversity indices	average value of pre-smog swabs	average value of post-smog swabs	P value (Paired t test)
Number of observed species	382.29±121.19	458.66±233.21	0.062
Shannon	5.28±0.68	5.35±0.50	0.526
Simpson	0.93±0.045	0.94±0.035	0.625
Chao1	488.19±206.98	590.81±351.96	0.132
ACE	480.59±143.99	616.37±372.80	0.036
Whole-tree phylogenetic diversity	149.24±100.66	149.37±76.05	0.993

Table S9. Comparison of alpha diversity indexes of pre- and post-smog pharyngeal microbiota people not wearing masks

Alpha diversity indices	average value of pre-smog swabs	average value of post-smog swabs	P value (Paired t test)
Number of observed species	376.47±120.31	505.96±245.24	0.001
Shannon	5.09±0.56	5.23±0.75	0.290
Simpson	0.92±0.04	0.93±0.58	0.511
Chao1	472.35±154.17	690.75±394.30	< 0.001
ACE	484.73±163.20	710.76±407.27	< 0.001
Whole-tree phylogenetic diversity	135.87±101.47	177.28±119.00	0.010

Table S10. Differences of influencing factors distribution among male and female

Influencing factors	Male	Female	Total	P value of χ^2 test
Working hours				P = 0.991
All day	23	26	49	
Morning	16	18	34	
With masks				P < 0.001
Yes	8	30	3	
No	31	14	45	
Smoking				P < 0.001

Yes	18	3	21	
No	21	41	62	
Using antibiotics within 30 days				P = 0.575
Yes	10	9	19	
No	29	35	64	
Having respiratory symptoms in last two weeks				P = 0.549
Yes	6	9	15	
No	33	35	68	

Table S11. The correlation between influencing factors and bacterial community structure of nasopharyngeal microbiota of 39 males

Influencing factors	RDA1	RDA2	r2	Pr (> r)
Smoggy event	1	0.00031	0.216268	0.0005
Working time	-0.63974	-0.76859	0.032288	0.305347
With masks	0.55302	0.833168	0.02109	0.46027
Smoking	-0.14759	-0.98905	0.191082	0.0005
Using antibiotics	-0.87514	-0.48387	0.105755	0.01949
Respiratory symptoms	0.342176	0.939636	0.070114	0.058971

Table S12. The genus list having positive correlation with influencing factors ($p < 0.05$)

Influencing factors	Genus	Source description	Human disease caused by this genus
Smog	*Corynebacterium_1*	Human	Local inflammation, systemic poisoning symptoms, myocardial and peripheral nerve damage
	Dolosigranulum	Human	Interstitial lung disease
	Leptotrichia	Human	Vaginitis, tissue necrosis, septicaemia, amniotic cavity infection, premature delivery, tropical ulcers
	unidentified_Chloroplast	Unknown	Unreported
	Peptoniphilus	Human	Rhinosinusitis
	Veillonella	Human, animal	Osteomyelitis, pneumonia, periodontitis
	Anaerococcus	Human	Vaginosis
	Gemella	Human, animal	Pneumonia, endophthalmitis, canaliculitis, endocarditis, bacteremia
	Staphylococcus	Human, animal, aquatic environment	Pneumonia, empyema, otitis media, meningitis, pericarditis, endocarditis, septicaemia, sepsis, etc.
	Granulicatella	Human	Endocarditis
	Ruminococcaceae_UCG-014	Unknown	Unreported
Smoking	*Staphylococcus*	Human, animal, aquatic environment	Pneumonia, empyema, otitis media, meningitis, pericarditis, endocarditis, septicaemia, sepsis, etc.

Actinobacillus	Human, animal, poultry	Endocarditis, pleuropneumoniae
Psychrobacter	Marine	Unreported
Peptoniphilus	Human	Rhinosinusitis
Actinomyces	Human, animal	Chronic abscess, multiple fistula, suppurative, swollen teeth, bone chyle, breast infection, etc.
Anaerococcus	Human	Vaginosis
Gemella	Human, animal	Pneumonia, endophthalmitis, canaliculitis, Endocarditis, bacteremia
Streptococcus	Human, animal, aquatic environment	Pneumonia, empyema, otitis media, meningitis, pericarditis, endocarditis, septicaemia, sepsis, etc.
*Treponema*_2	Human, animal	Unreported

Supplementary table 13. Changes of the five genera of respiratory pathogens in nasopharynx after exposure to smoggy days.

Genus or species	Relative abundance before smoggy days	Relative abundance after smoggy days	Pairwise sample correlation coefficient	*P* value of Paired *t* test
Neisseria	0.131121	0.108708	0.394	0.131
Streptococcus	0.074851	0.072699	0.415	0.735
Haemophilus	0.032924	0.036201	0.378	0.283
H. influenza	0.001709	0.003899	0.989	0.246
Moraxella	0.021857	0.029895	0.143	0.357
M. catarrhalis	0.017795	0.026480	0.130	0.314
Staphylococcus	0.001447	0.003900	0.109	0.065
